# Supplementary material for: Normative growth trajectories of fetal brain regions validated by satisfactory maturation of neurodevelopmental domains at 2 years of age
Source: Nat Commun. 2026 Feb 23;17:3073. doi: 10.1038/s41467-026-69657-w (PMC13040065; doi:10.1038/s41467-026-69657-w)
Supplement: Supplementary file 1 — Supplementary Information [file 41467_2026_69657_MOESM1_ESM.pdf]

Normative growth trajectories of fetal brain regions validated by satisfactory maturation of neurodevelopmental domains at 2 years of age

Supplementary Figures 1-9

1. Sensitivity analysis on INTER-NDA inclusion.
2. Fitted centiles (3rd, 50th and 97th) vs empirical data for volumetric trajectories.
3. Fitted centiles (3rd, 50th and 97th) vs empirical data for cortical growth trajectories.
4. Relative growth trajectories normalised by total brain volume (TBV).
5. Relative growth trajectories of each cortical lobe normalised by cortical plate volume (CoPV).
6. Feature importance in the Random Forest Model at 3 time points.
7. Hemisphere specific cortical plate growth trajectories.
8. Ultrasound properties
9. Deep learning networks segmentation performance on a held-out test set (n=70).

Supplementary Tables 1-12

1. Standardised differences between INTER-NDA (NDA) groups for each image derived phenotype (IDP).
2. Birth measures, and measures of neurodevelopmental outcomes at 2 years of age of infants included in the INTERGROWTH-21st Fetal Growth Longitudinal Study (FGLS) whose fetal brain volumes were analysed, compared to the total FGLS population.
3. Overview of the Fetal Growth Longitudinal Study (FGLS) 3D ultrasound (US) dataset.
4. Variance components analysis (VCA).
5. Standardised site differences (SSD) for each image derived phenotype (IDP).
6. Standardised Sex differences (SSD) for each image derived phenotype (IDP).
7. Standardised differences between cerebral hemispheres for each image derived phenotype (IDP)
8. Formulae for fetal brain growth trajectories
9. Cluster table summarising how the magnetic resonance imaging (MRI) cortical parcellation atlas labels were combined for comparison with the ultrasound-derived cortical labels
10. Demographic information about the training and testing dataset .
11. Summary of the magnetic resonance imaging (MRI) atlases used for label comparison.
12. Cluster table summarising how the magnetic resonance imaging atlas (MRI) labels were combined for comparison with the ultrasound atlas labels

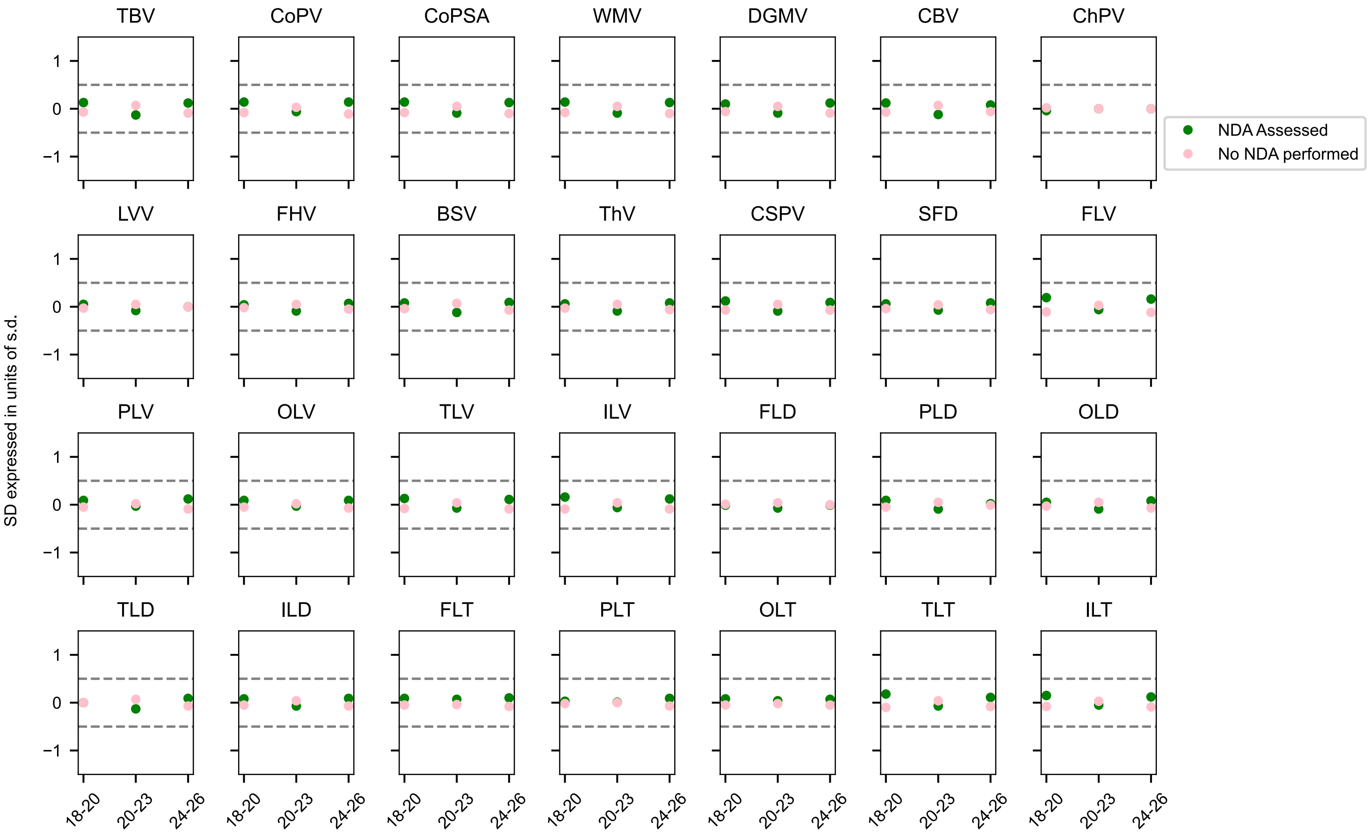

Supplementary Fig. 1. Sensitivity analysis on INTER-NDA inclusion. Standardised differences (SD) between those children who were (n=1,112) and were not (n=1,794) assessed at 2 years of age. Dotted lines indicate ±0.5 SD.

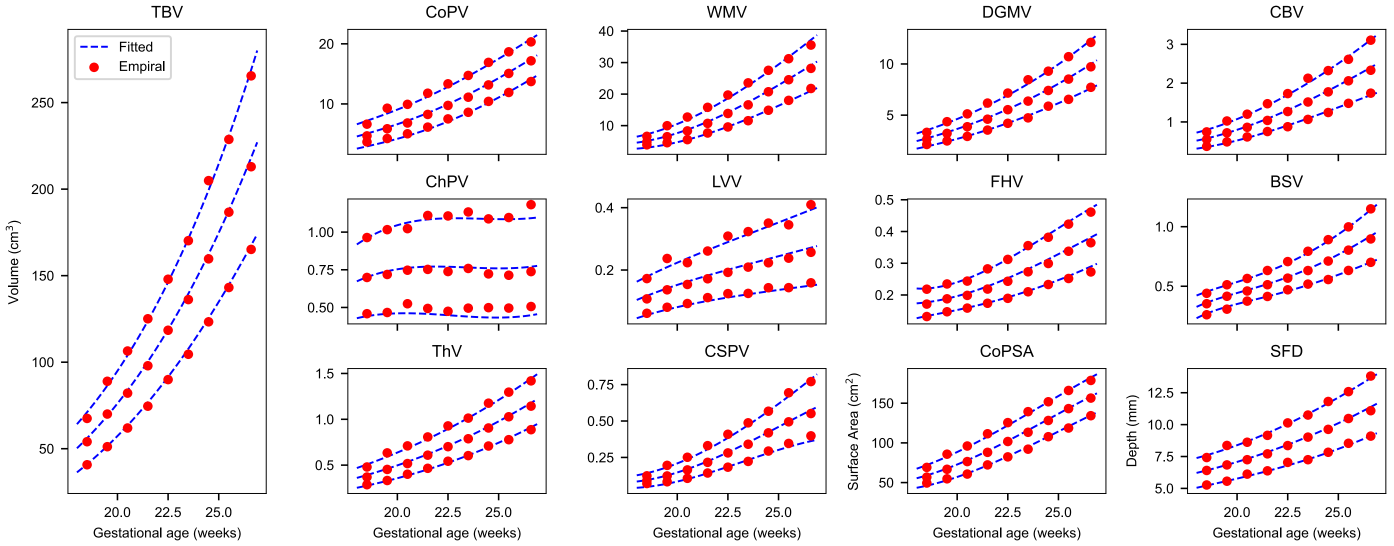

Supplementary Fig. 2. Fitted centiles (3rd, 50th and 97th) vs empirical data for volumetric trajectories. Trajectories showing total brain volume (TBV), cortical plate volume (CoPV), white matter volume (WMV), deep grey matter volume (DGMV), cerebellum volume (CBV), thalamus volume (ThV), lateral posterior ventricle horns volume (LVV), choroid plexus volume (ChPV), frontal horns volume (FHV), brainstem volume (BSV), cavum septum volume (CSPV), cortical plate surface area (CoPSA) and Sylvian fissure depth (SFD). For all structures except TBV and CSPV, the IDPs are measured only from the distal hemisphere. Supplementary Table 8 contains the number of subjects used for each plot.

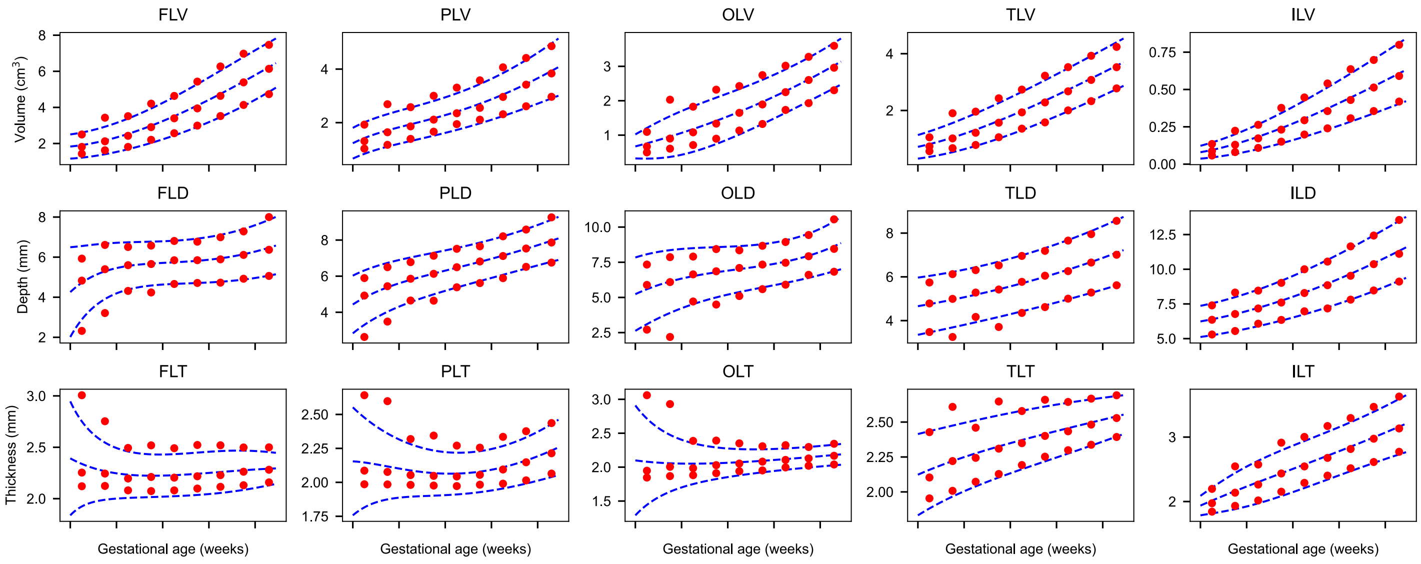

Supplementary Fig. 3. Fitted centiles (3rd, 50th and 97th) vs empirical data for cortical growth trajectories. These trajectories show frontal lobe volume (FLV), temporal lobe volume (TLV), parietal lobe volume (PLV), occipital lobe volume (OLV), insular lobe volume (ILV), frontal lobe depth (FLD), temporal lobe depth (TLD), parietal lobe depth (PLD), occipital lobe depth (OLD), insular lobe depth (ILD), frontal lobe thickness (FLT), temporal lobe thickness (TLT), parietal lobe thickness (PLT), occipital lobe thickness (OLT) and insular lobe thickness (ILT). Each of these IDPs is measured from the distal hemisphere. Supplementary Table 8 contains the number of subjects used for each plot.

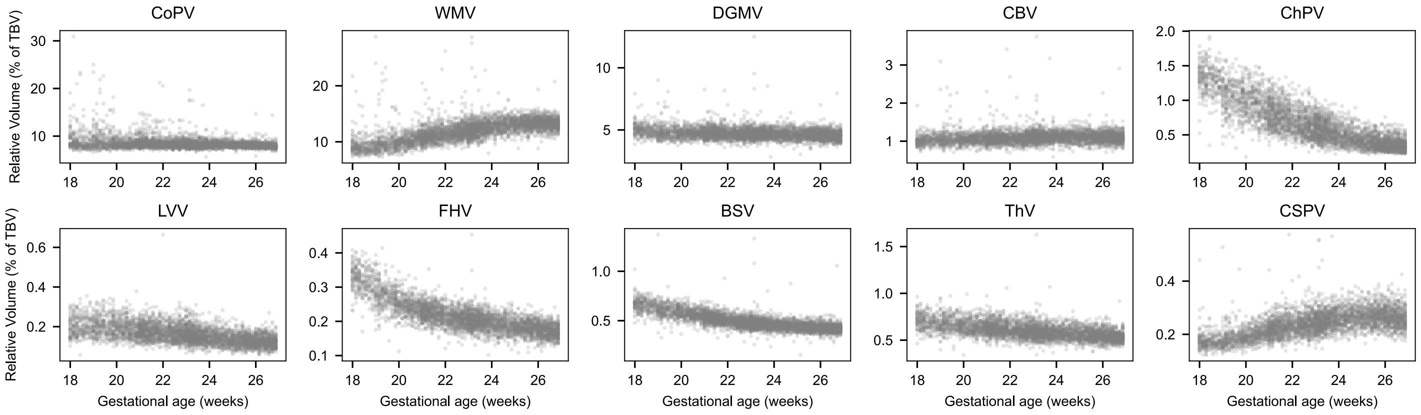

Supp Fig. 4. Relative growth trajectories normalised by total brain volume (TBV). Supplementary Table 8 contains the number of subjects used for each plot.

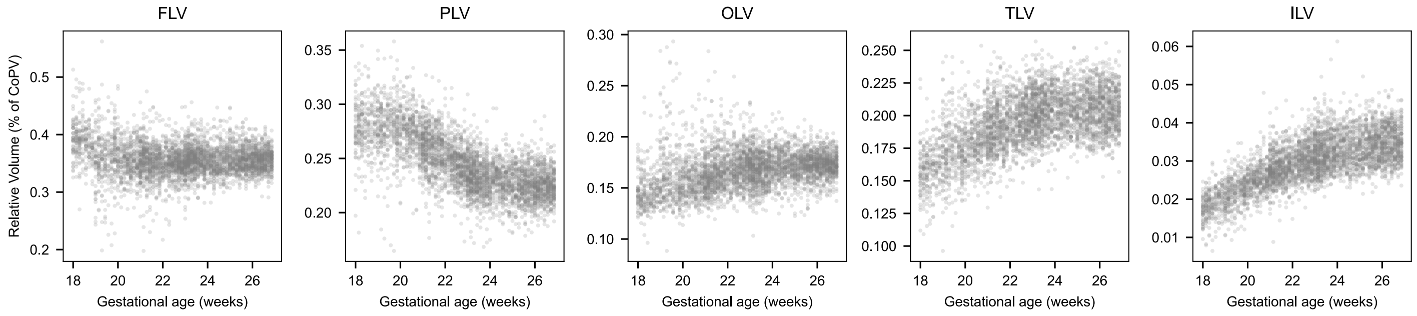

Supp Fig. 5. Relative growth trajectories of each cortical lobe normalised by cortical plate volume (CoPV). Supplementary Table 8 contains the number of subjects used for each plot.

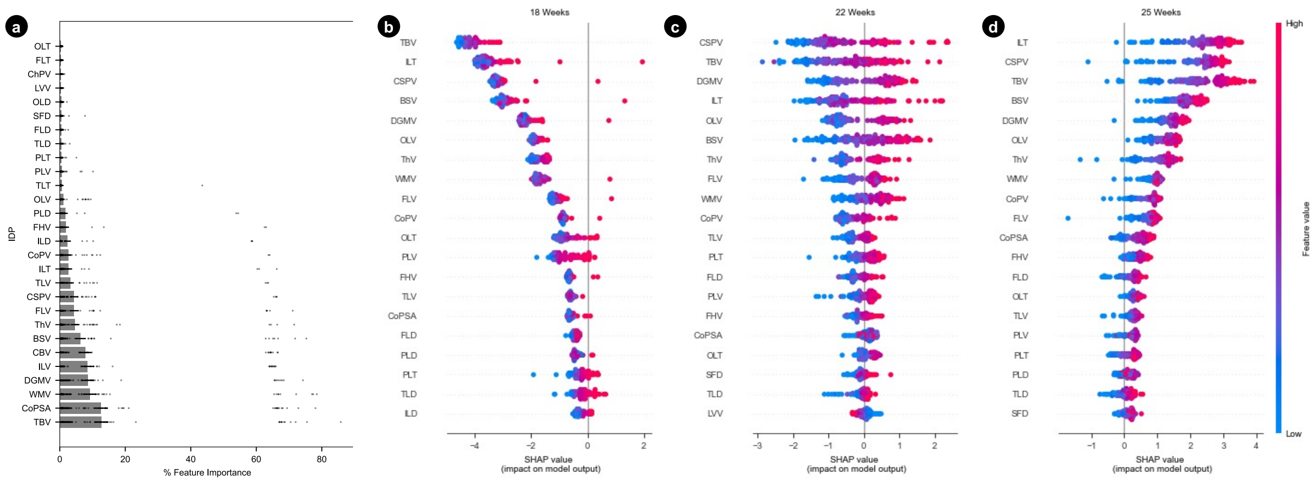

Supplementary Fig. 6. Feature importance in the Random Forest Model at 3 time points. (a) The importance of the 28 image-derived phenotypes (IDPs) for accurately predicting gestational age across the whole time period. Bars show the mean feature importance across all trees (100 trees) in the random forest, with error bars representing the standard error of the mean (SEM). The scatter shows the individual tree points. The relative importance of each image-derived phenotype (IDP) at 18 (b), 22 (c) and 26 (d) weeks' gestation. The top six most informative IDPs were consistent across these time periods. SHAP = SHapley Additive exPlanations.

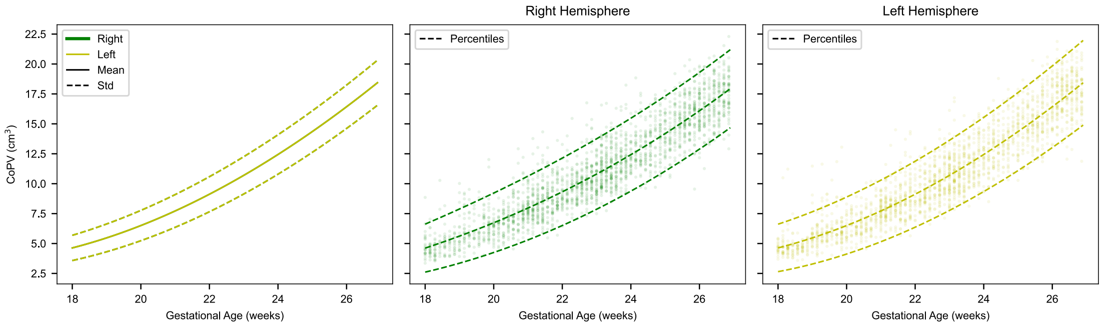

Supplementary Fig. 7. Hemisphere specific cortical plate growth trajectories. Growth model fitted for the left and right hemispheres separately. The two growth trajectories are indistinguishable from each other. These curves were computed using 4181 fetal brain ultrasound scans.

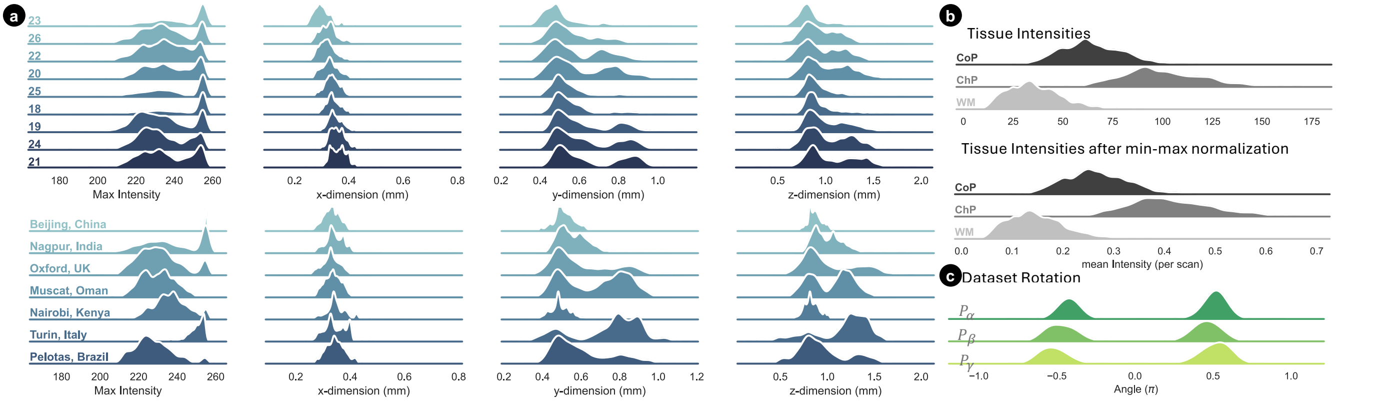

Supplementary Fig. 8. Ultrasound properties (a) An overview of the original ultrasound volumes properties across age and international site before the processing pipelines. Voxel resolution was determined by the ultrasound system settings and acquisition protocol, which were standardised across all sites and gestational ages as part of the INTERGROWTH-21st protocol (n=4205). (b) The tissue-intensities before and after normalization using the training dataset and atlas-propagated labels (n=404). (c) the rotation required to align each volume, showing the scanning protocol was consistent across the scans, with two peaks for forward and backwards facing fetuses (n=4205).

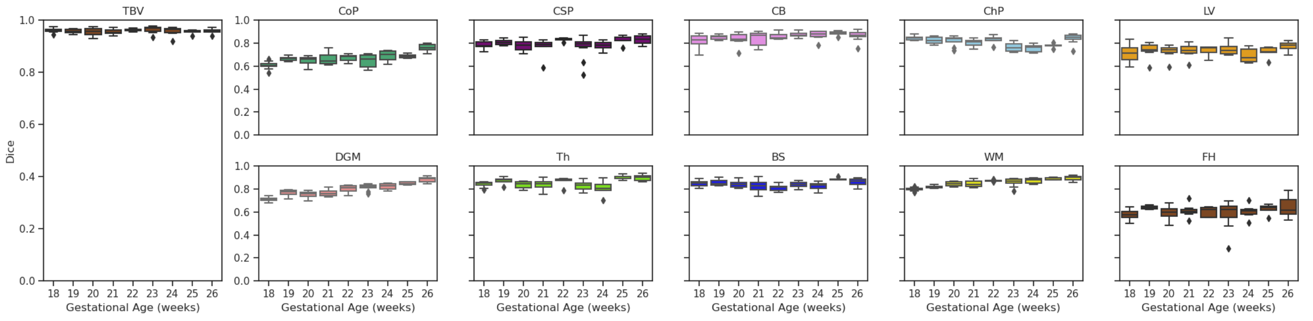

Supplementary Fig. 9. Deep learning networks segmentation performance on a held-out test set (n=70). Box plots show the distribution of Dice overlaps for each manually delineated structure at each gestational week. Each box extends from the frist quartile to the third and the ewhiskers show the farthest data point lyin within 1.5x the interquartile range. Structures evaluated include: total brain volume (TBV), cortical plate (CoP), white matter (WM), deep grey matter (DGM), cerebellum (CB), thalamus (Th), lateral posterior ventricle horns (LV), choroid plexus (ChP), frontal horns (FH), brainstem (BS) and cavum septum (CSP). The box spans the interquartile range (IQR, Q1-Q3), the line inside the box marks the median, whiskers extend to the most extreme data points within 1.5 x IQR, and points beyond are shown individually as outliers. Lobar parcellations (frontal, temporal, parietal, occipital, insular) were generated by diffeomorphic atlas-based template propagation and are therefore not included in these Dice metrics.

Supplementary Table 1- Standardised differences between INTER-NDA (NDA) groups for each image derived phenotype (IDP). Number of samples excluding outliers lying >4SD above the mean at each complete gestational age. The tables are separated by IDP: total brain volume (TBV), cortical plate volume (CoPV), white matter volume (WMV), deep grey matter volume (DGMV), cerebellum volume (CBV), thalamus volume (ThV), lateral ventricles volume (LVV), choroid plexus volume (ChPV), frontal horns volume (FHV), brainstem volume (BSV), cavum septum volume (CSPV), cortical plate suface area (CoPSA), Sylvian fissure depth (SFD), frontal lobe volume (FLV), temporal lobe volume (TLV), parietal lobe volume (PLV), occipital lobe volume (OLV), insular lobe volume (ILV), frontal lobe depth (FLD), temporal lobe depth (TLD), parietal lobe depth (PLD), occipital lobe depth (OLD), insular lobe depth (ILD), frontal lobe thickness (FLT), temporal lobe thickness (TLT), parietal lobe thickness (PLT), occipital lobe thickness (OLT) and insular lobe thickness (ILT).

GA (weeks)	NDA Status	Sample Total	Sample mean age	TBV Adj. mean	TBV Adj. SD	Standardised Effect
18-20	No NDA performed	633	19.47	69.04	14.59	-0.06
	Satisfactory NDA	324	19.62	71.71	16.2	0.12
	Pooled	957	19.52	69.94	15.2	0
21-23	No NDA performed	1157	22.58	120.96	22.2	0.08
	Satisfactory NDA	591	22.26	115.77	22.99	-0.15
	Pooled	1748	22.48	119.21	22.6	0
24-26	No NDA performed	869	25.48	186.72	31.71	-0.09
	Satisfactory NDA	622	25.63	194.04	33.01	0.13
	Pooled	1491	25.54	189.77	32.45	0

GA (weeks)	NDA Status	Sample Total	Sample mean age	CoPV Adj. mean	CoPV Adj. SD	Standardised Effect
18-20	No NDA performed	629	19.47	6	1.51	-0.07
	Satisfactory NDA	320	19.62	6.34	1.55	0.14
	Pooled	949	19.52	6.12	1.53	0
21-23	No NDA performed	1151	22.59	10.06	1.96	0.03
	Satisfactory NDA	589	22.27	9.89	2.06	-0.06
	Pooled	1740	22.48	10	2	0
24-26	No NDA performed	870	25.48	15.17	2.4	-0.1
	Satisfactory NDA	622	25.63	15.72	2.34	0.13
	Pooled	1492	25.54	15.4	2.39	0

GA (weeks)	NDA Status	Sample Total	Sample mean age	CoPSA Adj. mean	CoPSA Adj. SD	Standardised Effect
18-20	No NDA performed	628	19.47	67.78	11.04	-0.07
	Satisfactory NDA	320	19.62	69.98	11.56	0.13
	Pooled	948	19.52	68.52	11.26	0
21-23	No NDA performed	1155	22.59	103.4	15.38	0.05
	Satisfactory NDA	591	22.27	101.03	16.11	-0.1
	Pooled	1746	22.48	102.6	15.67	0
24-26	No NDA performed	870	25.48	142.63	16.29	-0.09
	Satisfactory NDA	622	25.63	146.34	16.19	0.13
	Pooled	1492	25.54	144.18	16.35	0

GA (weeks)	NDA Status	Sample Total	Sample mean age	WMV Adj. mean	WMV Adj. SD	Standardised Effect
18-20	No NDA performed	628	19.47	6.78	2.01	-0.06
	Satisfactory NDA	320	19.62	7.16	2.23	0.12
	Pooled	948	19.52	6.91	2.1	0

21-23	No NDA performed	1155	22.59	14.4	3.69	0.05
	Satisfactory NDA	591	22.27	13.83	3.88	-0.1
	Pooled	1746	22.48	14.21	3.76	0
24-26	No NDA performed	870	25.48	24.6	4.64	-0.09
	Satisfactory NDA	622	25.63	25.64	4.72	0.13
	Pooled	1492	25.54	25.04	4.7	0
GA (weeks)	NDA Status	Sample Total	Sample mean age	DGMV Adj. mean	DGMV Adj. SD	Standardised Effect
18-20	No NDA performed	630	19.47	3.33	0.69	-0.04
	Satisfactory NDA	322	19.62	3.41	0.74	0.07
	Pooled	952	19.52	3.35	0.71	0
21-23	No NDA performed	1155	22.59	5.68	1.08	0.06
	Satisfactory NDA	591	22.27	5.5	1.14	-0.11
	Pooled	1746	22.48	5.62	1.1	0
24-26	No NDA performed	870	25.48	8.58	1.41	-0.09
	Satisfactory NDA	621	25.63	8.88	1.45	0.12
	Pooled	1491	25.54	8.71	1.43	0
GA (weeks)	NDA Status	Sample Total	Sample mean age	CBV Adj. mean	CBV Adj. SD	Standardised Effect
18-20	No NDA performed	630	19.47	0.72	0.19	-0.05
	Satisfactory NDA	320	19.62	0.75	0.19	0.1
	Pooled	950	19.52	0.73	0.19	0
21-23	No NDA performed	1152	22.58	1.33	0.3	0.07
	Satisfactory NDA	589	22.27	1.26	0.32	-0.14
	Pooled	1741	22.48	1.3	0.31	0
24-26	No NDA performed	870	25.48	2.08	0.39	-0.05
	Satisfactory NDA	620	25.63	2.13	0.41	0.07
	Pooled	1490	25.54	2.1	0.4	0
GA (weeks)	NDA Status	Sample Total	Sample mean age	ChPV Adj. mean	ChPV Adj. SD	Standardised Effect
18-20	No NDA performed	634	19.47	0.73	0.15	0.03
	Satisfactory NDA	328	19.61	0.72	0.14	-0.06
	Pooled	962	19.52	0.73	0.15	0
21-23	No NDA performed	1159	22.59	0.77	0.18	0
	Satisfactory NDA	592	22.27	0.77	0.17	0
	Pooled	1751	22.48	0.77	0.18	0
24-26	No NDA performed	869	25.48	0.75	0.17	0
	Satisfactory NDA	621	25.63	0.75	0.17	0
	Pooled	1490	25.54	0.75	0.17	0
GA (weeks)	NDA Status	Sample Total	Sample mean age	LVV Adj. mean	LVV Adj. SD	Standardised Effect
18-20	No NDA performed	634	19.47	0.14	0.04	-0.02
	Satisfactory NDA	327	19.61	0.14	0.04	0.04
	Pooled	961	19.52	0.14	0.04	0
21-23	No NDA performed	1158	22.59	0.2	0.05	0.05
	Satisfactory NDA	592	22.27	0.19	0.05	-0.1
	Pooled	1750	22.48	0.2	0.05	0

24-26	No NDA performed	870	25.48	0.25	0.06	0.01
	Satisfactory NDA	622	25.63	0.25	0.06	-0.02
	Pooled	1492	25.54	0.25	0.06	0
GA (weeks)	NDA Status	Sample Total	Sample mean age	FHV Adj. mean	FHV Adj. SD	Standardised Effect
18-20	No NDA performed	634	19.47	0.19	0.03	-0.02
	Satisfactory NDA	326	19.62	0.19	0.03	0.04
	Pooled	960	19.52	0.19	0.03	0
21-23	No NDA performed	1157	22.58	0.25	0.04	0.06
	Satisfactory NDA	592	22.27	0.24	0.04	-0.12
	Pooled	1749	22.48	0.25	0.04	0
24-26	No NDA performed	870	25.48	0.34	0.05	-0.06
	Satisfactory NDA	622	25.63	0.34	0.05	0.08
	Pooled	1492	25.54	0.34	0.05	0
GA (weeks)	NDA Status	Sample Total	Sample mean age	BSV Adj. mean	BSV Adj. SD	Standardised Effect
18-20	No NDA performed	633	19.47	0.41	0.07	-0.03
	Satisfactory NDA	324	19.62	0.42	0.07	0.06
	Pooled	957	19.52	0.42	0.07	0
21-23	No NDA performed	1155	22.58	0.58	0.08	0.06
	Satisfactory NDA	591	22.27	0.57	0.09	-0.12
	Pooled	1746	22.48	0.58	0.08	0
24-26	No NDA performed	869	25.48	0.81	0.13	-0.07
	Satisfactory NDA	621	25.64	0.83	0.13	0.1
	Pooled	1490	25.54	0.82	0.13	0
GA (weeks)	NDA Status	Sample Total	Sample mean age	ThV Adj. mean	ThV Adj. SD	Standardised Effect
18-20	No NDA performed	634	19.47	0.46	0.1	-0.02
	Satisfactory NDA	325	19.62	0.47	0.1	0.04
	Pooled	959	19.52	0.46	0.1	0
21-23	No NDA performed	1157	22.58	0.72	0.12	0.05
	Satisfactory NDA	590	22.27	0.7	0.13	-0.1
	Pooled	1747	22.48	0.71	0.13	0
24-26	No NDA performed	870	25.48	1.03	0.16	-0.06
	Satisfactory NDA	621	25.63	1.06	0.17	0.09
	Pooled	1491	25.54	1.05	0.16	0
GA (weeks)	NDA Status	Sample Total	Sample mean age	CSPV Adj. mean	CSPV Adj. SD	Standardised Effect
18-20	No NDA performed	633	19.47	0.13	0.04	-0.05
	Satisfactory NDA	322	19.62	0.14	0.05	0.09
	Pooled	955	19.52	0.13	0.04	0
21-23	No NDA performed	1156	22.59	0.29	0.08	0.05
	Satisfactory NDA	590	22.27	0.28	0.08	-0.11
	Pooled	1746	22.48	0.29	0.08	0
24-26	No NDA performed	870	25.48	0.5	0.11	-0.06
	Satisfactory NDA	621	25.63	0.51	0.11	0.09
	Pooled	1491	25.54	0.5	0.11	0

GA (weeks)	NDA Status	Sample Total	Sample mean age	SFD Adj. mean	SFD Adj. SD	Standardised Effect
18-20	No NDA performed	632	19.47	6.85	0.76	-0.05
	Satisfactory NDA	324	19.62	6.95	0.84	0.09
	Pooled	956	19.52	6.88	0.79	0
21-23	No NDA performed	1149	22.58	8.47	1.01	0.04
	Satisfactory NDA	590	22.27	8.34	1.04	-0.08
	Pooled	1739	22.48	8.43	1.02	0
24-26	No NDA performed	868	25.48	10.45	1.29	-0.08
	Satisfactory NDA	620	25.63	10.71	1.28	0.12
	Pooled	1488	25.54	10.56	1.29	0
GA (weeks)	NDA Status	Sample Total	Sample mean age	FLV Adj. mean	FLV Adj. SD	Standardised Effect
18-20	No NDA performed	631	19.47	2.17	0.47	-0.08
	Satisfactory NDA	319	19.63	2.29	0.49	0.17
	Pooled	950	19.53	2.21	0.48	0
21-23	No NDA performed	1149	22.59	3.55	0.74	0.03
	Satisfactory NDA	588	22.27	3.48	0.74	-0.06
	Pooled	1737	22.48	3.52	0.74	0
24-26	No NDA performed	870	25.48	5.38	0.93	-0.11
	Satisfactory NDA	622	25.63	5.63	0.91	0.16
	Pooled	1492	25.54	5.49	0.93	0
GA (weeks)	NDA Status	Sample Total	Sample mean age	PLV Adj. mean	PLV Adj. SD	Standardised Effect
18-20	No NDA performed	625	19.47	1.65	0.39	-0.06
	Satisfactory NDA	322	19.62	1.73	0.41	0.13
	Pooled	947	19.52	1.68	0.4	0
21-23	No NDA performed	1149	22.59	2.43	0.4	0.02
	Satisfactory NDA	588	22.27	2.41	0.45	-0.03
	Pooled	1737	22.48	2.42	0.41	0
24-26	No NDA performed	869	25.48	3.44	0.6	-0.08
	Satisfactory NDA	622	25.63	3.55	0.6	0.11
	Pooled	1491	25.54	3.49	0.6	0
GA (weeks)	NDA Status	Sample Total	Sample mean age	OLV Adj. mean	OLV Adj. SD	Standardised Effect
18-20	No NDA performed	624	19.47	0.94	0.33	-0.07
	Satisfactory NDA	322	19.62	1.01	0.37	0.14
	Pooled	946	19.52	0.97	0.34	0
21-23	No NDA performed	1152	22.59	1.69	0.41	0.01
	Satisfactory NDA	590	22.27	1.68	0.44	-0.02
	Pooled	1742	22.48	1.69	0.42	0
24-26	No NDA performed	869	25.48	2.63	0.44	-0.05
	Satisfactory NDA	622	25.63	2.69	0.45	0.08
	Pooled	1491	25.54	2.65	0.44	0
GA (weeks)	NDA Status	Sample Total	Sample mean age	TLV Adj. mean	TLV Adj. SD	Standardised Effect
18-20	No NDA performed	628	19.48	1.04	0.33	-0.08
	Satisfactory NDA	321	19.62	1.12	0.36	0.15

	Pooled	949	19.53	1.06	0.34	0
21-23	No NDA performed	1153	22.59	2	0.48	0.04
	Satisfactory NDA	589	22.27	1.95	0.5	-0.07
	Pooled	1742	22.48	1.98	0.49	0
24-26	No NDA performed	869	25.48	3.11	0.53	-0.08
	Satisfactory NDA	622	25.63	3.21	0.52	0.11
	Pooled	1491	25.54	3.15	0.53	0
GA (weeks)	NDA Status	Sample Total	Sample mean age	ILV Adj. mean	ILV Adj. SD	Standardised Effect
18-20	No NDA performed	630	19.47	0.13	0.05	-0.07
	Satisfactory NDA	320	19.62	0.14	0.05	0.13
	Pooled	950	19.52	0.14	0.05	0
21-23	No NDA performed	1155	22.59	0.31	0.09	0.04
	Satisfactory NDA	588	22.27	0.3	0.09	-0.07
	Pooled	1743	22.48	0.31	0.09	0
24-26	No NDA performed	870	25.48	0.52	0.11	-0.09
	Satisfactory NDA	620	25.64	0.54	0.11	0.13
	Pooled	1490	25.54	0.53	0.12	0
GA (weeks)	NDA Status	Sample Total	Sample mean age	FLD Adj. mean	FLD Adj. SD	Standardised Effect
18-20	No NDA performed	634	19.47	5.18	0.88	-0.01
	Satisfactory NDA	325	19.62	5.2	0.93	0.01
	Pooled	959	19.52	5.19	0.89	0
21-23	No NDA performed	1146	22.59	5.79	0.56	0.07
	Satisfactory NDA	588	22.26	5.66	0.63	-0.14
	Pooled	1734	22.48	5.75	0.59	0
24-26	No NDA performed	867	25.48	6.16	0.71	-0.02
	Satisfactory NDA	620	25.63	6.2	0.73	0.03
	Pooled	1487	25.54	6.18	0.72	0
GA (weeks)	NDA Status	Sample Total	Sample mean age	PLD Adj. mean	PLD Adj. SD	Standardised Effect
18-20	No NDA performed	627	19.47	5.3	0.85	-0.06
	Satisfactory NDA	324	19.62	5.43	0.87	0.11
	Pooled	951	19.52	5.34	0.86	0
21-23	No NDA performed	1147	22.59	6.5	0.65	0.07
	Satisfactory NDA	585	22.27	6.36	0.71	-0.14
	Pooled	1732	22.48	6.45	0.67	0
24-26	No NDA performed	868	25.48	7.53	0.65	-0.03
	Satisfactory NDA	620	25.63	7.58	0.71	0.05
	Pooled	1488	25.54	7.55	0.67	0
GA (weeks)	NDA Status	Sample Total	Sample mean age	OLD Adj. mean	OLD Adj. SD	Standardised Effect
18-20	No NDA performed	633	19.47	6.05	1.22	-0.01
	Satisfactory NDA	327	19.61	6.11	1.3	0.03
	Pooled	960	19.52	6.07	1.25	0
21-23	No NDA performed	1150	22.58	7.08	0.91	0.06
	Satisfactory NDA	589	22.27	6.92	0.92	-0.11

	Pooled	1739	22.48	7.03	0.92	0
24-26	No NDA performed	866	25.48	7.96	0.93	-0.08
	Satisfactory NDA	621	25.63	8.15	1.04	0.11
	Pooled	1487	25.54	8.04	0.98	0
GA (weeks)	NDA Status	Sample Total	Sample mean age	TLD Adj. mean	TLD Adj. SD	Standardised Effect
18-20	No NDA performed	628	19.47	4.98	0.66	0
	Satisfactory NDA	323	19.62	4.97	0.77	-0.01
	Pooled	951	19.53	4.98	0.7	0
21-23	No NDA performed	1153	22.59	5.77	0.74	0.07
	Satisfactory NDA	586	22.27	5.61	0.75	-0.14
	Pooled	1739	22.48	5.71	0.75	0
24-26	No NDA performed	866	25.48	6.6	0.79	-0.11
	Satisfactory NDA	622	25.63	6.81	0.86	0.15
	Pooled	1488	25.54	6.69	0.83	0
GA (weeks)	NDA Status	Sample Total	Sample mean age	ILD Adj. mean	ILD Adj. SD	Standardised Effect
18-20	No NDA performed	631	19.47	6.78	0.72	-0.05
	Satisfactory NDA	325	19.62	6.89	0.82	0.09
	Pooled	956	19.52	6.82	0.75	0
21-23	No NDA performed	1151	22.58	8.36	0.98	0.04
	Satisfactory NDA	590	22.27	8.23	0.99	-0.08
	Pooled	1741	22.48	8.31	0.98	0
24-26	No NDA performed	868	25.48	10.38	1.27	-0.09
	Satisfactory NDA	620	25.63	10.65	1.25	0.12
	Pooled	1488	25.54	10.49	1.27	0
GA (weeks)	NDA Status	Sample Total	Sample mean age	FLT Adj. mean	FLT Adj. SD	Standardised Effect
18-20	No NDA performed	628	19.47	2.27	0.17	-0.06
	Satisfactory NDA	324	19.62	2.3	0.19	0.11
	Pooled	952	19.52	2.28	0.18	0
21-23	No NDA performed	1151	22.59	2.23	0.11	-0.03
	Satisfactory NDA	584	22.27	2.24	0.11	0.06
	Pooled	1735	22.48	2.23	0.11	0
24-26	No NDA performed	869	25.48	2.27	0.1	-0.06
	Satisfactory NDA	619	25.63	2.28	0.1	0.08
	Pooled	1488	25.54	2.27	0.1	0
GA (weeks)	NDA Status	Sample Total	Sample mean age	PLT Adj. mean	PLT Adj. SD	Standardised Effect
18-20	No NDA performed	628	19.47	2.11	0.14	0
	Satisfactory NDA	323	19.62	2.11	0.14	0
	Pooled	951	19.52	2.11	0.14	0
21-23	No NDA performed	1144	22.59	2.07	0.08	-0.02
	Satisfactory NDA	587	22.27	2.08	0.09	0.05
	Pooled	1731	22.48	2.07	0.08	0
24-26	No NDA performed	870	25.48	2.16	0.11	-0.06
	Satisfactory NDA	621	25.63	2.18	0.1	0.08

	Pooled	1491	25.54	2.17	0.11	0
GA (weeks)	NDA Status	Sample Total	Sample mean age	OLT Adj. mean	OLT Adj. SD	Standardised Effect
18-20	No NDA performed	630	19.47	2.06	0.25	-0.02
	Satisfactory NDA	320	19.62	2.08	0.27	0.04
	Pooled	950	19.52	2.06	0.26	0
21-23	No NDA performed	1148	22.59	2.07	0.1	-0.03
	Satisfactory NDA	585	22.27	2.08	0.12	0.06
	Pooled	1733	22.48	2.08	0.11	0
24-26	No NDA performed	869	25.48	2.15	0.08	-0.04
	Satisfactory NDA	622	25.63	2.15	0.09	0.05
	Pooled	1491	25.54	2.15	0.09	0
GA (weeks)	NDA Status	Sample Total	Sample mean age	TLT Adj. mean	TLT Adj. SD	Standardised Effect
18-20	No NDA performed	630	19.47	2.2	0.14	-0.11
	Satisfactory NDA	324	19.61	2.24	0.15	0.21
	Pooled	954	19.52	2.21	0.14	0
21-23	No NDA performed	1153	22.58	2.37	0.12	0.03
	Satisfactory NDA	589	22.27	2.36	0.12	-0.05
	Pooled	1742	22.48	2.37	0.12	0
24-26	No NDA performed	870	25.48	2.48	0.09	-0.08
	Satisfactory NDA	621	25.64	2.5	0.09	0.11
	Pooled	1491	25.54	2.49	0.09	0
GA (weeks)	NDA Status	Sample Total	Sample mean age	ILT Adj. mean	ILT Adj. SD	Standardised Effect
18-20	No NDA performed	629	19.47	2.15	0.18	-0.06
	Satisfactory NDA	321	19.62	2.18	0.18	0.12
	Pooled	950	19.52	2.16	0.18	0
21-23	No NDA performed	1149	22.58	2.59	0.22	0.01
	Satisfactory NDA	590	22.27	2.58	0.23	-0.03
	Pooled	1739	22.47	2.58	0.22	0
24-26	No NDA performed	870	25.48	2.98	0.25	-0.09
	Satisfactory NDA	620	25.64	3.04	0.25	0.12
	Pooled	1490	25.54	3.01	0.25	0

Supplementary Table 2 - Birth measures, and measures of neurodevelopmental outcomes at 2 years of age of infants included in the INTERGROWTH-21st Fetal Growth Longitudinal Study (FGLS) whose fetal brain volumes were analysed, compared to the total FGLS population.

	**INTERGROWTH-21st fetal brain volume analysed**	**INTERGROWTH-21st total FGLS population**
**Newborns measured**	n=2805	n=4321
Mean ± SD		
Weight (kg)	3.2 (0.5)	3.3 (0.4)
Length (cm)	49.1 (2.0)	49.4 (1.9)
Head circumference (cm)	33.7 (1.3)	33.9 (1.3)
**Infants measured at age 2**	n=2523	n=3042
Mean ± SD		
Weight (z-score)	0.3 (1.1)	0.2 (1.1)
Height (z-score)	0.1 (1.1)	0.0 (1.1)
Head circumference (z-score)	0.0 (1.1)	0.0 (1.1)
**Infants assessed with the INTER-NDA at age 2**	n=1011	n=1181
Mean (normative range)		
Cognitive[1]	75.8 (28.2-100)	71.8 (38.5-99.6)
Language[1]	68.4 (12.1-100)	63.5 (17.8-100)
Fine motor[1]	83.2 (25.0-100)	78.5 (25.7-100)
Gross motor[1]	84.2 (33.3-100)	79.6 (51.7-100)
Emotional affect, Median (IQR) and Mean (normative range)		
Positive affect[1]	90 (70-100)	90 (51.4-100)
Negative affect[2]	0 (0-25)	25 (0.0-76.5)
Vision, Median (IQR)		
Visual Acuity (LogMAR)	0.2 (0.1-0.2)	0.2 (0.1-0.2)
Contrast sensitivity (%)	1.5 (1.0-1.5)	1.5 (1.0-2.0)

[1] For these domains, higher scores reflect better outcome
[2] For negative behaviour, lower scores reflect better outcomes

Supplementary Table 3: Overview of the Fetal Growth Longitudinal Study (FGLS) 3D ultrasound (US) dataset. A breakdown of the number of fetuses and US scans included in our analysis. Includes contribution per study site, sex, age, and an overview of the longitudinal data.

**FGLS fetuses included:**	
Entire FGLS dataset	4321
Fetuses with available 3D scans	4141
Fetuses with follow-up and without severe morbidity	3556
Fetuses excluding those who scored in the bottom 3% on any one of the INTER-NDA domains, or had low scores for visual acuity at 2 years of age	2906
Fetuses (number of scans) with scan within gestational age range 18 to 27 weeks	2805 (4205)
**Fetus (scan) count per sex:**	
Female	1417 (2145)
Male	1388 (2060)
**Fetus (scan) count per study site:**	
Pelotas, Brazil	330 (520)
Beijing, China	479 (596)
Nagpur, India	527 (821)
Turin, Italy	314 (474)
Nairobi, Kenya	399 (571)
Muscat, Oman	437 (759)
Oxford, UK	319 (464)
**Number of scans at each gestational week (right hemisphere):**	
18	272 (133)
19	326 (146)
20	364 (210)
21	571 (296)
22	501 (261)
23	679 (361)
24	414 (215)
25	496 (268)
26	582 (301)
**Number of scans per fetus:**	
1	1410
2	1390
3	5
Mean (STD) number of scans:	1.50 (0.5)
Median number of scans:	1.0
**Average interval between scans:**	
Mean (STD) interval:	4.61 (0.58) weeks

Median interval:	5.0 weeks
**Count of each interval**	
3 weeks	5
4 weeks	603
5 weeks	741
6 weeks	46
7 weeks	3
8 weeks	2
**Average intervals per fetus:**	
Average mean interval per fetus:	4.91 weeks
Average median interval per fetus:	4.91 weeks
Smallest interval across fetuses:	3.9 weeks
Largest interval across fetuses:	8.7 weeks

Supplementary Table 4: Variance components analysis (VCA). VCA for total brain volume (TBV), cortical plate volume (CoPV), white matter volume (WMV), deep grey matter volume (DGMV), cerebellum volume (CBV), thalamus volume (ThV), lateral posterior ventricle horns volume (LVV), choroid plexus volume (ChPV), frontal horns volume (FHV), brainstem volume (BSV), cavum septum volume (CSPV), cortical plate surface area (CoPSA), Sylvian fissure depth (SFD), frontal lobe volume (FLV), temporal lobe volume (TLV), parietal lobe volume (PLV), occipital lobe volume (OLV), insular lobe volume (ILV), frontal lobe depth (FLD), temporal lobe depth (TLD), parietal lobe depth (PLD), occipital lobe depth (OLD), insular lobe depth (ILD), frontal lobe thickness (FLT), temporal lobe thickness (TLT), parietal lobe thickness (PLT), occipital lobe thickness (OLT) and insular lobe thickness (ILT).

Brain Structure	Between-site variance (%)	Within-site variance (%)	Residual variance (%)
TBV	1.6	11.9	86.5
CoPV	4.4	8.9	86.7
WMV	3.0	12.9	84.1
DGMV	2.9	8.5	88.6
CBV	0.8	6.0	93.1
ChPV	0.7	2.1	97.1
LVV	0.6	0.8	98.7
FHV	1.0	6.1	92.9
BSV	0.9	7.4	91.7
ThV	1.9	4.6	93.5
CSPV	3.0	7.1	89.9
CoPSA	3.8	4.8	91.4
SFD	2.4	3.2	94.4
FLV	2.2	10.5	87.2
PLV	5.8	6.3	88.0
OLV	5.4	5.6	89.0
TLV	2.6	5.3	92.2
ILV	1.3	4.9	93.8
FLD	1.2	5.1	93.7
PLD	0.6	1.6	97.8
OLD	1.9	3.1	95.0
TLD	2.2	0.9	96.9
ILD	2.1	4.9	93.0
FLT	1.3	8.6	90.0
PLT	2.0	18.7	79.3
OLT	0.9	8.0	91.1
TLT	0.9	4.6	94.5
ILT	1.9	1.9	96.2

Supplementary Table 5 - Standardised site differences (SSD) for each image derived phenotype (IDP). Number of samples excluding outliers lying >4SD above the mean at each complete gestational age (GA). The tables are separated by IDP: total brain volume (TBV), cortical plate volume (CoPV), white matter volume (WMV), deep grey matter volume (DGMV), cerebellum volume (CBV), thalamus volume (ThV), lateral ventricles volume (LVV), choroid plexus volume (ChPV), frontal horns volume (FHV), brainstem volume (BSV), cavum septum volume (CSPV), cortical plate suface area (CoPSA), Sylvian fissure depth (SFD), frontal lobe volume (FLV), temporal lobe volume (TLV), parietal lobe volume (PLV), occipital lobe volume (OLV), insular lobe volume (ILV), frontal lobe depth (FLD), temporal lobe depth (TLD), parietal lobe depth (PLD), occipital lobe depth (OLD), insular lobe depth (ILD), frontal lobe thickness (FLT), temporal lobe thickness (TLT), parietal lobe thickness (PLT), occipital lobe thickness (OLT) and insular lobe thickness (ILT).

GA (weeks)	Study site	Sample Total	Sample mean GA	TBV Adj. mean	TBV Adj. SD	Standardised Effect
18-20	Pelotas (Brazil)	130	19.73	73.69	14.00	0.25
	Beijing (China)	117	18.49	54.66	8.28	-1.01
	Nagpur (India)	184	19.46	68.21	14.26	-0.11
	Nairobi (Kenya)	133	19.49	71.87	15.51	0.13
	Muscat (Oman)	219	19.89	74.84	13.39	0.32
	Oxford (UK)	96	19.30	65.29	15.06	-0.31
	Turin (Italy)	78	20.17	79.42	13.22	0.62
	**All Sites**	957	19.52	69.94	15.20	0.00
21-23	Pelotas (Brazil)	181	22.26	116.61	22.25	-0.11
	Beijing (China)	436	23.05	128.76	17.90	0.42
	Nagpur (India)	330	22.41	115.35	21.80	-0.17
	Nairobi (Kenya)	236	22.48	123.90	23.35	0.21
	Muscat (Oman)	210	22.11	111.42	22.12	-0.34
	Oxford (UK)	187	22.49	119.22	22.84	0.00
	Turin (Italy)	168	21.78	107.92	23.80	-0.50
	**All Sites**	1748	22.48	119.21	22.60	0.00
24-26	Pelotas (Brazil)	209	25.49	190.23	29.54	0.01
	Beijing (China)	41	24.54	159.56	27.57	-0.93
	Nagpur (India)	304	25.56	188.48	34.17	-0.04
	Nairobi (Kenya)	200	25.42	188.45	33.16	-0.04
	Muscat (Oman)	330	25.45	184.80	30.07	-0.15
	Oxford (UK)	180	25.60	190.21	33.00	0.01
	Turin (Italy)	227	25.95	204.60	29.41	0.46
	**All Sites**	1491	25.54	189.77	32.45	0.00

GA (weeks)	Study site	Sample Total	Sample mean GA	CoPV Adj. mean	CoPV Adj. SD	Standardised Effect
18-20	Pelotas (Brazil)	130	19.73	6.27	1.42	0.10
	Beijing (China)	118	18.49	4.83	1.07	-0.84
	Nagpur (India)	181	19.46	6.16	1.44	0.03
	Nairobi (Kenya)	130	19.50	6.54	1.64	0.28
	Muscat (Oman)	218	19.89	6.42	1.41	0.20
	Oxford (UK)	94	19.29	5.67	1.65	-0.29
	Turin (Italy)	78	20.17	6.68	1.31	0.37
	**All Sites**	949	19.52	6.12	1.53	0.00
21-23	Pelotas (Brazil)	180	22.27	9.86	2.06	-0.07
	Beijing (China)	435	23.05	10.38	1.59	0.19
	Nagpur (India)	330	22.41	9.79	1.84	-0.10
	Nairobi (Kenya)	233	22.49	10.77	2.13	0.38
	Muscat (Oman)	210	22.11	9.42	1.94	-0.29
	Oxford (UK)	184	22.50	10.13	2.17	0.07
	Turin (Italy)	168	21.78	9.10	2.22	-0.45
	**All Sites**	1740	22.48	10.00	2.00	0.00
24-26	Pelotas (Brazil)	209	25.49	15.27	2.22	-0.05
	Beijing (China)	41	24.54	12.70	2.18	-1.13
	Nagpur (India)	304	25.56	15.35	2.40	-0.02
	Nairobi (Kenya)	200	25.42	15.61	2.53	0.09
	Muscat (Oman)	330	25.45	15.03	2.26	-0.15
	Oxford (UK)	180	25.60	15.29	2.44	-0.04
	Turin (Italy)	228	25.95	16.47	2.02	0.45
	**All Sites**	1492	25.54	15.40	2.39	0.00

GA (weeks)	Study site	Sample Total	Sample mean GA	WMV Adj. mean	WMV Adj. SD	Standardised Effect
18-20	Pelotas (Brazil)	130	19.73	7.16	1.96	0.12
	Beijing (China)	117	18.49	4.92	0.71	-0.95
	Nagpur (India)	181	19.46	6.73	1.86	-0.09
	Nairobi (Kenya)	130	19.50	7.41	2.28	0.24
	Muscat (Oman)	218	19.89	7.51	1.98	0.29
	Oxford (UK)	94	19.29	6.26	1.98	-0.31
	Turin (Italy)	78	20.17	8.14	2.13	0.59
	**All Sites**	948	19.52	6.91	2.10	0.00
21-23	Pelotas (Brazil)	180	22.27	13.62	3.66	-0.16
	Beijing (China)	436	23.05	15.27	3.02	0.28
	Nagpur (India)	330	22.41	13.69	3.56	-0.14
	Nairobi (Kenya)	236	22.49	15.61	4.12	0.37
	Muscat (Oman)	210	22.11	12.97	3.59	-0.33

	Oxford (UK)	186	22.50	14.48	4.00	0.07
	Turin (Italy)	168	21.78	12.37	3.91	-0.49
	**All Sites**	1746	22.48	14.21	3.76	0.00
24-26	Pelotas (Brazil)	209	25.49	24.54	4.34	-0.11
	Beijing (China)	41	24.54	19.76	4.46	-1.12
	Nagpur (India)	304	25.56	25.08	4.84	0.01
	Nairobi (Kenya)	200	25.42	25.42	4.76	0.08
	Muscat (Oman)	330	25.45	24.56	4.52	-0.10
	Oxford (UK)	180	25.60	24.94	4.83	-0.02
	Turin (Italy)	228	25.95	26.81	4.04	0.38
	**All Sites**	1492	25.54	25.04	4.70	0.00

GA (weeks)	Study site	Sample Total	Sample mean GA	DGMV Adj. mean	DGMV Adj. SD	Standardised Effect
18-20	Pelotas (Brazil)	130	19.73	3.40	0.63	0.07
	Beijing (China)	117	18.49	2.68	0.32	-0.96
	Nagpur (India)	182	19.46	3.25	0.64	-0.14
	Nairobi (Kenya)	131	19.50	3.52	0.71	0.24
	Muscat (Oman)	219	19.89	3.58	0.67	0.33
	Oxford (UK)	95	19.30	3.17	0.72	-0.26
	Turin (Italy)	78	20.17	3.81	0.69	0.65
	**All Sites**	952	19.52	3.35	0.71	0.00
21-23	Pelotas (Brazil)	180	22.27	5.40	1.09	-0.20
	Beijing (China)	435	23.05	6.00	0.90	0.35
	Nagpur (India)	330	22.41	5.41	1.04	-0.19
	Nairobi (Kenya)	235	22.49	6.00	1.14	0.35
	Muscat (Oman)	210	22.11	5.24	1.05	-0.34
	Oxford (UK)	188	22.50	5.68	1.14	0.06
	Turin (Italy)	168	21.78	5.13	1.18	-0.44
	**All Sites**	1746	22.48	5.62	1.10	0.00
24-26	Pelotas (Brazil)	208	25.49	8.57	1.30	-0.09
	Beijing (China)	41	24.54	7.19	1.37	-1.06
	Nagpur (India)	304	25.56	8.68	1.49	-0.02
	Nairobi (Kenya)	200	25.42	8.78	1.44	0.05
	Muscat (Oman)	330	25.45	8.50	1.36	-0.15
	Oxford (UK)	180	25.60	8.76	1.45	0.04
	Turin (Italy)	228	25.95	9.35	1.25	0.45
	**All Sites**	1491	25.54	8.71	1.43	0.00

GA (weeks)	Study site	Sample Total	Sample mean GA	CBV Adj. mean	CBV Adj. SD	Standardised Effect
18-20	Pelotas (Brazil)	130	19.73	0.76	0.17	0.16
	Beijing (China)	118	18.49	0.56	0.12	-0.94
	Nagpur (India)	181	19.46	0.72	0.16	-0.07
	Nairobi (Kenya)	131	19.50	0.75	0.19	0.07
	Muscat (Oman)	219	19.89	0.79	0.18	0.32
	Oxford (UK)	93	19.29	0.69	0.20	-0.26
	Turin (Italy)	78	20.17	0.85	0.18	0.62
	**All Sites**	950	19.52	0.73	0.19	0.00
21-23	Pelotas (Brazil)	180	22.27	1.24	0.29	-0.20
	Beijing (China)	434	23.05	1.42	0.25	0.38
	Nagpur (India)	330	22.41	1.26	0.30	-0.15
	Nairobi (Kenya)	232	22.49	1.36	0.33	0.19
	Muscat (Oman)	210	22.11	1.21	0.28	-0.31
	Oxford (UK)	187	22.50	1.34	0.33	0.10
	Turin (Italy)	168	21.78	1.16	0.32	-0.46
	**All Sites**	1741	22.48	1.30	0.31	0.00
24-26	Pelotas (Brazil)	209	25.49	2.08	0.37	-0.06
	Beijing (China)	41	24.54	1.80	0.34	-0.77
	Nagpur (India)	304	25.56	2.06	0.40	-0.12
	Nairobi (Kenya)	198	25.41	2.10	0.40	0.00
	Muscat (Oman)	330	25.45	2.08	0.37	-0.06
	Oxford (UK)	180	25.60	2.14	0.39	0.09
	Turin (Italy)	228	25.95	2.25	0.40	0.37
	**All Sites**	1490	25.54	2.10	0.40	0.00

GA (weeks)	Study site	Sample Total	Sample mean GA	ChPV Adj. mean	ChPV Adj. SD	Standardised Effect
18-20	Pelotas (Brazil)	130	19.73	0.73	0.15	0.02
	Beijing (China)	118	18.49	0.70	0.14	-0.19
	Nagpur (India)	187	19.46	0.70	0.14	-0.22
	Nairobi (Kenya)	134	19.48	0.73	0.14	0.03
	Muscat (Oman)	219	19.89	0.74	0.14	0.10
	Oxford (UK)	96	19.30	0.74	0.15	0.09
	Turin (Italy)	78	20.17	0.78	0.15	0.35
	**All Sites**	962	19.52	0.73	0.15	0.00
21-23	Pelotas (Brazil)	181	22.26	0.75	0.18	-0.12
	Beijing (China)	437	23.05	0.80	0.18	0.17

	Nagpur (India)	330	22.41	0.75	0.17	-0.11
	Nairobi (Kenya)	237	22.49	0.76	0.17	-0.05
	Muscat (Oman)	210	22.11	0.76	0.16	-0.03
	Oxford (UK)	188	22.50	0.77	0.18	-0.01
	Turin (Italy)	168	21.78	0.78	0.17	0.04
	**All Sites**	1751	22.48	0.77	0.18	0.00
24-26	Pelotas (Brazil)	208	25.49	0.74	0.17	-0.04
	Beijing (China)	41	24.54	0.79	0.14	0.24
	Nagpur (India)	304	25.56	0.74	0.17	-0.07
	Nairobi (Kenya)	199	25.41	0.75	0.17	0.00
	Muscat (Oman)	330	25.45	0.73	0.15	-0.11
	Oxford (UK)	180	25.60	0.77	0.18	0.09
	Turin (Italy)	228	25.95	0.78	0.18	0.17
	**All Sites**	1490	25.54	0.75	0.17	0.00

GA (weeks)	Study site	Sample Total	Sample mean GA	LVV Adj. mean	LVV Adj. SD	Standardised Effect
18-20	Pelotas (Brazil)	130	19.73	0.14	0.04	0.07
	Beijing (China)	118	18.49	0.11	0.03	-0.56
	Nagpur (India)	186	19.46	0.13	0.04	-0.11
	Nairobi (Kenya)	134	19.48	0.15	0.04	0.21
	Muscat (Oman)	219	19.89	0.14	0.04	0.14
	Oxford (UK)	96	19.30	0.13	0.04	-0.10
	Turin (Italy)	78	20.17	0.15	0.04	0.38
	**All Sites**	961	19.52	0.14	0.04	0.00
21-23	Pelotas (Brazil)	181	22.26	0.19	0.05	-0.06
	Beijing (China)	437	23.05	0.22	0.05	0.37
	Nagpur (India)	330	22.41	0.19	0.05	-0.15
	Nairobi (Kenya)	237	22.49	0.20	0.05	0.03
	Muscat (Oman)	210	22.11	0.18	0.04	-0.27
	Oxford (UK)	187	22.50	0.20	0.05	-0.02
	Turin (Italy)	168	21.78	0.18	0.05	-0.28
	**All Sites**	1750	22.48	0.20	0.05	0.00
24-26	Pelotas (Brazil)	209	25.49	0.24	0.06	-0.16
	Beijing (China)	41	24.54	0.25	0.08	0.08
	Nagpur (India)	304	25.56	0.25	0.06	-0.03
	Nairobi (Kenya)	200	25.42	0.24	0.07	-0.13
	Muscat (Oman)	330	25.45	0.25	0.06	-0.02

	Oxford (UK)	180	25.60	0.25	0.06	0.04
	Turin (Italy)	228	25.95	0.27	0.06	0.29
	**All Sites**	1492	25.54	0.25	0.06	0.00

GA (weeks)	Study site	Sample Total	Sample mean GA	FHV Adj. mean	FHV Adj. SD	Standardised Effect
18-20	Pelotas (Brazil)	130	19.73	0.19	0.02	-0.05
	Beijing (China)	118	18.49	0.18	0.02	-0.39
	Nagpur (India)	185	19.47	0.18	0.02	-0.16
	Nairobi (Kenya)	134	19.48	0.19	0.03	-0.02
	Muscat (Oman)	219	19.89	0.19	0.02	0.18
	Oxford (UK)	96	19.30	0.19	0.03	-0.09
	Turin (Italy)	78	20.17	0.20	0.03	0.67
	**All Sites**	960	19.52	0.19	0.03	0.00
21-23	Pelotas (Brazil)	181	22.26	0.24	0.04	-0.20
	Beijing (China)	435	23.05	0.27	0.04	0.39
	Nagpur (India)	330	22.41	0.24	0.04	-0.20
	Nairobi (Kenya)	237	22.49	0.26	0.05	0.19
	Muscat (Oman)	210	22.11	0.24	0.04	-0.31
	Oxford (UK)	188	22.50	0.25	0.04	0.06
	Turin (Italy)	168	21.78	0.23	0.04	-0.36
	**All Sites**	1749	22.48	0.25	0.04	0.00
24-26	Pelotas (Brazil)	209	25.49	0.33	0.05	-0.11
	Beijing (China)	41	24.54	0.30	0.06	-0.83
	Nagpur (India)	304	25.56	0.34	0.05	-0.07
	Nairobi (Kenya)	200	25.42	0.34	0.05	-0.03
	Muscat (Oman)	330	25.45	0.34	0.05	-0.08
	Oxford (UK)	180	25.60	0.34	0.05	0.08
	Turin (Italy)	228	25.95	0.36	0.05	0.42
	**All Sites**	1492	25.54	0.34	0.05	0.00

GA (weeks)	Study site	Sample Total	Sample mean GA	BSV Adj. mean	BSV Adj. SD	Standardised Effect
18-20	Pelotas (Brazil)	130	19.73	0.44	0.06	0.28
	Beijing (China)	118	18.49	0.36	0.05	-0.89

	Nagpur (India)	185	19.46	0.41	0.07	-0.07
	Nairobi (Kenya)	132	19.50	0.42	0.07	0.07
	Muscat (Oman)	219	19.89	0.44	0.06	0.28
	Oxford (UK)	95	19.30	0.39	0.07	-0.33
	Turin (Italy)	78	20.17	0.45	0.06	0.54
	**All Sites**	957	19.52	0.42	0.07	0.00
21-23	Pelotas (Brazil)	181	22.26	0.57	0.09	-0.14
	Beijing (China)	435	23.05	0.61	0.07	0.35
	Nagpur (India)	330	22.41	0.57	0.08	-0.13
	Nairobi (Kenya)	235	22.49	0.59	0.09	0.10
	Muscat (Oman)	210	22.11	0.56	0.07	-0.28
	Oxford (UK)	187	22.50	0.58	0.09	0.06
	Turin (Italy)	168	21.78	0.55	0.09	-0.36
	**All Sites**	1746	22.48	0.58	0.08	0.00
24-26	Pelotas (Brazil)	208	25.50	0.82	0.12	0.00
	Beijing (China)	41	24.54	0.70	0.10	-0.94
	Nagpur (India)	304	25.56	0.83	0.13	0.04
	Nairobi (Kenya)	199	25.42	0.80	0.12	-0.17
	Muscat (Oman)	330	25.45	0.81	0.12	-0.08
	Oxford (UK)	180	25.60	0.82	0.13	0.03
	Turin (Italy)	228	25.95	0.86	0.13	0.35
	**All Sites**	1490	25.54	0.82	0.13	0.00

GA (weeks)	Study site	Sample Total	Sample mean GA	ThV Adj. mean	ThV Adj. SD	Standardised Effect
18-20	Pelotas (Brazil)	130	19.73	0.47	0.09	0.08
	Beijing (China)	118	18.49	0.37	0.06	-0.92
	Nagpur (India)	185	19.46	0.45	0.09	-0.10
	Nairobi (Kenya)	133	19.49	0.47	0.09	0.12
	Muscat (Oman)	219	19.89	0.49	0.09	0.33
	Oxford (UK)	96	19.30	0.45	0.10	-0.17
	Turin (Italy)	78	20.17	0.52	0.09	0.58
	**All Sites**	959	19.52	0.46	0.10	0.00
21-23	Pelotas (Brazil)	181	22.26	0.69	0.13	-0.17
	Beijing (China)	436	23.05	0.74	0.11	0.26
	Nagpur (India)	330	22.41	0.70	0.12	-0.14
	Nairobi (Kenya)	236	22.49	0.74	0.13	0.23
	Muscat (Oman)	210	22.11	0.69	0.12	-0.21

	Oxford (UK)	186	22.49	0.72	0.13	0.10
	Turin (Italy)	168	21.78	0.66	0.13	-0.39
	**All Sites**	1747	22.48	0.71	0.13	0.00
24-26	Pelotas (Brazil)	209	25.49	1.03	0.16	-0.09
	Beijing (China)	41	24.54	0.87	0.15	-1.04
	Nagpur (India)	304	25.56	1.04	0.17	-0.02
	Nairobi (Kenya)	199	25.41	1.03	0.16	-0.07
	Muscat (Oman)	330	25.45	1.04	0.16	-0.03
	Oxford (UK)	180	25.60	1.05	0.16	0.02
	Turin (Italy)	228	25.95	1.11	0.15	0.38
	**All Sites**	1491	25.54	1.05	0.16	0.00

GA (weeks)	Study site	Sample Total	Sample mean GA	CSPV Adj. mean	CSPV Adj. SD	Standardised Effect
18-20	Pelotas (Brazil)	130	19.73	0.14	0.04	0.08
	Beijing (China)	117	18.49	0.09	0.01	-0.95
	Nagpur (India)	184	19.46	0.13	0.04	-0.11
	Nairobi (Kenya)	132	19.50	0.14	0.05	0.21
	Muscat (Oman)	219	19.89	0.15	0.04	0.31
	Oxford (UK)	95	19.30	0.12	0.04	-0.30
	Turin (Italy)	78	20.17	0.16	0.05	0.69
	**All Sites**	955	19.52	0.13	0.04	0.00
21-23	Pelotas (Brazil)	181	22.26	0.27	0.08	-0.24
	Beijing (China)	436	23.05	0.31	0.07	0.27
	Nagpur (India)	330	22.41	0.28	0.07	-0.15
	Nairobi (Kenya)	233	22.49	0.32	0.09	0.42
	Muscat (Oman)	210	22.11	0.27	0.08	-0.23
	Oxford (UK)	188	22.50	0.29	0.08	0.07
	Turin (Italy)	168	21.78	0.25	0.08	-0.50
	**All Sites**	1746	22.48	0.29	0.08	0.00
24-26	Pelotas (Brazil)	209	25.49	0.49	0.09	-0.13
	Beijing (China)	41	24.54	0.38	0.09	-1.12
	Nagpur (India)	303	25.56	0.50	0.10	-0.04
	Nairobi (Kenya)	200	25.42	0.51	0.11	0.09
	Muscat (Oman)	330	25.45	0.50	0.11	-0.04
	Oxford (UK)	180	25.60	0.51	0.11	0.09
	Turin (Italy)	228	25.95	0.53	0.09	0.29
	**All Sites**	1491	25.54	0.50	0.11	0.00

GA (weeks)	Study site	Sample Total	Sample mean GA	CoPSA Adj. mean	CoPSA Adj. SD	Standardised Effect
18-20	Pelotas (Brazil)	130	19.73	69.80	10.49	0.11
	Beijing (China)	117	18.49	57.49	5.30	-0.98
	Nagpur (India)	181	19.46	67.99	10.10	-0.05
	Nairobi (Kenya)	130	19.50	71.52	11.51	0.27
	Muscat (Oman)	218	19.89	71.63	10.51	0.28
	Oxford (UK)	94	19.29	64.96	11.61	-0.32
	Turin (Italy)	78	20.17	74.81	10.57	0.56
	**All Sites**	948	19.52	68.52	11.26	0.00
21-23	Pelotas (Brazil)	180	22.27	100.29	15.64	-0.15
	Beijing (China)	436	23.05	107.39	12.43	0.31
	Nagpur (India)	330	22.41	100.21	14.79	-0.15
	Nairobi (Kenya)	236	22.49	108.38	16.41	0.37
	Muscat (Oman)	210	22.11	97.22	14.99	-0.34
	Oxford (UK)	186	22.50	103.72	16.58	0.07
	Turin (Italy)	168	21.78	94.68	16.72	-0.51
	**All Sites**	1746	22.48	102.60	15.67	0.00
24-26	Pelotas (Brazil)	209	25.49	142.91	14.88	-0.08
	Beijing (China)	41	24.54	125.35	16.60	-1.15
	Nagpur (India)	304	25.56	144.01	16.83	-0.01
	Nairobi (Kenya)	200	25.42	144.99	16.43	0.05
	Muscat (Oman)	330	25.45	142.05	15.85	-0.13
	Oxford (UK)	180	25.60	144.07	16.80	-0.01
	Turin (Italy)	228	25.95	151.39	13.32	0.44
	**All Sites**	1492	25.54	144.18	16.35	0.00

GA (weeks)	Study site	Sample Total	Sample mean GA	SFD Adj. mean	SFD Adj. SD	Standardised Effect
18-20	Pelotas (Brazil)	130	19.73	7.15	0.77	0.34
	Beijing (China)	118	18.49	6.39	0.68	-0.62
	Nagpur (India)	184	19.46	6.73	0.82	-0.20
	Nairobi (Kenya)	131	19.50	6.84	0.74	-0.05
	Muscat (Oman)	219	19.89	7.08	0.73	0.25
	Oxford (UK)	96	19.30	6.71	0.78	-0.21

	Turin (Italy)	78	20.17	7.26	0.63	0.48
	**All Sites**	956	19.52	6.88	0.79	0.00
21-23	Pelotas (Brazil)	180	22.27	8.46	1.05	0.03
	Beijing (China)	434	23.05	8.72	0.92	0.29
	Nagpur (India)	330	22.41	8.25	0.97	-0.18
	Nairobi (Kenya)	233	22.48	8.47	1.11	0.04
	Muscat (Oman)	210	22.11	8.14	0.99	-0.28
	Oxford (UK)	184	22.49	8.51	0.98	0.08
	Turin (Italy)	168	21.78	8.20	1.07	-0.22
	**All Sites**	1739	22.48	8.43	1.02	0.00
24-26	Pelotas (Brazil)	209	25.49	10.74	1.11	0.14
	Beijing (China)	41	24.54	9.47	0.97	-0.84
	Nagpur (India)	304	25.56	10.44	1.27	-0.09
	Nairobi (Kenya)	199	25.41	10.36	1.45	-0.16
	Muscat (Oman)	329	25.45	10.34	1.21	-0.17
	Oxford (UK)	180	25.60	10.59	1.21	0.02
	Turin (Italy)	226	25.95	11.22	1.26	0.51
	**All Sites**	1488	25.54	10.56	1.29	0.00

GA (weeks)	Study site	Sample Total	Sample mean GA	FLV Adj. mean	FLV Adj. SD	Standardised Effect
18-20	Pelotas (Brazil)	130	19.73	2.30	0.48	0.18
	Beijing (China)	117	18.50	1.86	0.33	-0.74
	Nagpur (India)	182	19.47	2.27	0.52	0.12
	Nairobi (Kenya)	130	19.50	2.33	0.53	0.25
	Muscat (Oman)	218	19.89	2.26	0.42	0.09
	Oxford (UK)	95	19.30	2.05	0.45	-0.33
	Turin (Italy)	78	20.17	2.33	0.45	0.25
	**All Sites**	950	19.53	2.21	0.48	0.00
21-23	Pelotas (Brazil)	180	22.27	3.46	0.73	-0.08
	Beijing (China)	434	23.05	3.69	0.63	0.23
	Nagpur (India)	328	22.42	3.47	0.71	-0.07
	Nairobi (Kenya)	232	22.50	3.70	0.75	0.24
	Muscat (Oman)	210	22.11	3.29	0.73	-0.31
	Oxford (UK)	186	22.49	3.59	0.79	0.09
	Turin (Italy)	167	21.78	3.23	0.80	-0.40
	**All Sites**	1737	22.48	3.52	0.74	0.00
	Pelotas (Brazil)	209	25.49	5.53	0.90	0.05

24-26	Beijing (China)	41	24.54	4.53	0.84	-1.03
	Nagpur (India)	304	25.56	5.47	0.90	-0.02
	Nairobi (Kenya)	200	25.42	5.50	0.97	0.02
	Muscat (Oman)	330	25.45	5.32	0.89	-0.18
	Oxford (UK)	180	25.60	5.46	0.94	-0.03
	Turin (Italy)	228	25.95	5.89	0.81	0.44
	**All Sites**	1492	25.54	5.49	0.93	0.00

GA (weeks)	Study site	Sample Total	Sample mean GA	PLV Adj. mean	PLV Adj. SD	Standardised Effect
18-20	Pelotas (Brazil)	129	19.72	1.72	0.37	0.09
	Beijing (China)	118	18.49	1.34	0.27	-0.86
	Nagpur (India)	181	19.46	1.67	0.35	-0.03
	Nairobi (Kenya)	130	19.49	1.79	0.45	0.28
	Muscat (Oman)	217	19.89	1.77	0.36	0.22
	Oxford (UK)	94	19.30	1.58	0.43	-0.26
	Turin (Italy)	78	20.17	1.85	0.33	0.44
	**All Sites**	947	19.52	1.68	0.40	0.00
21-23	Pelotas (Brazil)	179	22.27	2.42	0.44	-0.02
	Beijing (China)	435	23.05	2.45	0.33	0.07
	Nagpur (India)	330	22.41	2.39	0.36	-0.08
	Nairobi (Kenya)	232	22.49	2.60	0.48	0.43
	Muscat (Oman)	210	22.11	2.36	0.40	-0.16
	Oxford (UK)	183	22.49	2.40	0.44	-0.05
	Turin (Italy)	168	21.78	2.27	0.46	-0.37
	**All Sites**	1737	22.48	2.42	0.41	0.00
24-26	Pelotas (Brazil)	209	25.49	3.40	0.56	-0.14
	Beijing (China)	41	24.54	2.88	0.48	-1.01
	Nagpur (India)	304	25.56	3.47	0.60	-0.02
	Nairobi (Kenya)	199	25.42	3.62	0.68	0.22
	Muscat (Oman)	330	25.45	3.41	0.54	-0.12
	Oxford (UK)	180	25.60	3.44	0.60	-0.07
	Turin (Italy)	228	25.95	3.72	0.56	0.38
	**All Sites**	1491	25.54	3.49	0.60	0.00

GA (weeks)	Study site	Sample Total	Sample mean GA	OLV Adj. mean	OLV Adj. SD	Standardised Effect
18-20	Pelotas (Brazil)	129	19.72	0.96	0.29	-0.02
	Beijing (China)	117	18.49	0.71	0.18	-0.75

	Nagpur (India)	183	19.46	0.99	0.38	0.07
	Nairobi (Kenya)	131	19.50	1.09	0.41	0.37
	Muscat (Oman)	216	19.89	1.02	0.31	0.17
	Oxford (UK)	92	19.31	0.86	0.32	-0.32
	Turin (Italy)	78	20.17	1.06	0.29	0.27
	**All Sites**	946	19.52	0.97	0.34	0.00
21-23	Pelotas (Brazil)	180	22.27	1.68	0.48	-0.03
	Beijing (China)	436	23.05	1.73	0.34	0.10
	Nagpur (India)	330	22.41	1.64	0.38	-0.12
	Nairobi (Kenya)	234	22.48	1.90	0.44	0.50
	Muscat (Oman)	210	22.11	1.60	0.42	-0.22
	Oxford (UK)	184	22.51	1.70	0.45	0.03
	Turin (Italy)	168	21.78	1.50	0.44	-0.44
	**All Sites**	1742	22.48	1.69	0.42	0.00
24-26	Pelotas (Brazil)	209	25.49	2.59	0.43	-0.15
	Beijing (China)	41	24.54	2.19	0.41	-1.05
	Nagpur (India)	304	25.56	2.63	0.47	-0.05
	Nairobi (Kenya)	199	25.41	2.71	0.46	0.13
	Muscat (Oman)	330	25.45	2.64	0.40	-0.04
	Oxford (UK)	180	25.60	2.63	0.46	-0.06
	Turin (Italy)	228	25.95	2.82	0.37	0.38
	**All Sites**	1491	25.54	2.65	0.44	0.00

GA (weeks)	Study site	Sample Total	Sample mean GA	TLV Adj. mean	TLV Adj. SD	Standardised Effect
18-20	Pelotas (Brazil)	130	19.73	1.08	0.31	0.05
	Beijing (China)	118	18.49	0.76	0.20	-0.88
	Nagpur (India)	182	19.46	1.08	0.35	0.06
	Nairobi (Kenya)	130	19.50	1.13	0.36	0.20
	Muscat (Oman)	218	19.89	1.15	0.33	0.24
	Oxford (UK)	93	19.31	0.96	0.33	-0.31
	Turin (Italy)	78	20.17	1.23	0.31	0.48
	**All Sites**	949	19.53	1.06	0.34	0.00
21-23	Pelotas (Brazil)	180	22.27	1.93	0.50	-0.11
	Beijing (China)	435	23.05	2.10	0.38	0.23

	Nagpur (India)	330	22.41	1.92	0.46	-0.14
	Nairobi (Kenya)	233	22.50	2.15	0.53	0.35
	Muscat (Oman)	210	22.11	1.84	0.45	-0.30
	Oxford (UK)	186	22.50	2.06	0.54	0.16
	Turin (Italy)	168	21.78	1.75	0.51	-0.48
	**All Sites**	1742	22.48	1.98	0.49	0.00
24-26	Pelotas (Brazil)	208	25.50	3.13	0.49	-0.03
	Beijing (China)	41	24.54	2.59	0.52	-1.06
	Nagpur (India)	304	25.56	3.16	0.52	0.02
	Nairobi (Kenya)	200	25.42	3.17	0.53	0.03
	Muscat (Oman)	330	25.45	3.07	0.51	-0.16
	Oxford (UK)	180	25.60	3.14	0.53	-0.03
	Turin (Italy)	228	25.95	3.37	0.47	0.42
	**All Sites**	1491	25.54	3.15	0.53	0.00

GA (weeks)	Study site	Sample Total	Sample mean GA	ILV Adj. mean	ILV Adj. SD	Standardised Effect
18-20	Pelotas (Brazil)	130	19.73	0.15	0.05	0.20
	Beijing (China)	117	18.49	0.09	0.02	-0.97
	Nagpur (India)	182	19.46	0.13	0.04	-0.08
	Nairobi (Kenya)	131	19.50	0.15	0.06	0.18
	Muscat (Oman)	218	19.89	0.15	0.05	0.26
	Oxford (UK)	94	19.29	0.12	0.05	-0.26
	Turin (Italy)	78	20.17	0.17	0.05	0.60
	**All Sites**	950	19.52	0.14	0.05	0.00
21-23	Pelotas (Brazil)	180	22.27	0.30	0.09	-0.11
	Beijing (China)	435	23.05	0.33	0.08	0.25
	Nagpur (India)	329	22.41	0.30	0.09	-0.10
	Nairobi (Kenya)	235	22.50	0.33	0.09	0.24
	Muscat (Oman)	210	22.11	0.28	0.08	-0.32
	Oxford (UK)	187	22.49	0.32	0.10	0.14
	Turin (Italy)	167	21.78	0.27	0.09	-0.42
	**All Sites**	1743	22.48	0.31	0.09	0.00
24-26	Pelotas (Brazil)	208	25.49	0.52	0.11	-0.05
	Beijing (China)	41	24.54	0.43	0.09	-0.81
	Nagpur (India)	304	25.56	0.53	0.12	0.04
	Nairobi (Kenya)	200	25.42	0.52	0.11	-0.06
	Muscat (Oman)	330	25.45	0.50	0.11	-0.18

	Oxford (UK)	179	25.61	0.53	0.12	0.07
	Turin (Italy)	228	25.95	0.57	0.11	0.40
	**All Sites**	1490	25.54	0.53	0.12	0.00

GA (weeks)	Study site	Sample Total	Sample mean GA	FLD Adj. mean	FLD Adj. SD	Standardised Effect
18-20	Pelotas (Brazil)	130	19.73	5.39	0.72	0.23
	Beijing (China)	118	18.49	4.70	1.00	-0.55
	Nagpur (India)	186	19.47	5.06	1.00	-0.15
	Nairobi (Kenya)	133	19.48	5.08	1.01	-0.12
	Muscat (Oman)	219	19.89	5.45	0.66	0.30
	Oxford (UK)	95	19.30	5.06	0.90	-0.15
	Turin (Italy)	78	20.17	5.50	0.58	0.35
	**All Sites**	959	19.52	5.19	0.89	0.00
21-23	Pelotas (Brazil)	180	22.27	5.77	0.67	0.04
	Beijing (China)	432	23.05	5.90	0.51	0.26
	Nagpur (India)	329	22.41	5.67	0.59	-0.14
	Nairobi (Kenya)	234	22.49	5.67	0.69	-0.13
	Muscat (Oman)	209	22.12	5.70	0.56	-0.08
	Oxford (UK)	184	22.49	5.70	0.60	-0.07
	Turin (Italy)	166	21.77	5.69	0.50	-0.09
	**All Sites**	1734	22.48	5.75	0.59	0.00
24-26	Pelotas (Brazil)	209	25.49	6.25	0.70	0.09
	Beijing (China)	41	24.54	6.02	0.54	-0.22
	Nagpur (India)	304	25.56	6.12	0.72	-0.09
	Nairobi (Kenya)	196	25.43	6.08	0.80	-0.13
	Muscat (Oman)	330	25.45	6.13	0.64	-0.06
	Oxford (UK)	180	25.60	6.14	0.69	-0.05
	Turin (Italy)	227	25.95	6.41	0.79	0.32
	**All Sites**	1487	25.54	6.18	0.72	0.00

GA (weeks)	Study site	Sample Total	Sample mean GA	PLD Adj. mean	PLD Adj. SD	Standardised Effect
18-20	Pelotas (Brazil)	130	19.73	5.56	0.69	0.25
	Beijing (China)	118	18.49	4.80	0.92	-0.63
	Nagpur (India)	182	19.47	5.32	0.90	-0.03
	Nairobi (Kenya)	130	19.47	5.39	0.94	0.05
	Muscat (Oman)	218	19.89	5.53	0.71	0.22
	Oxford (UK)	95	19.30	5.10	0.88	-0.29

	Turin (Italy)	78	20.17	5.55	0.72	0.24
	**All Sites**	951	19.52	5.34	0.86	0.00
21-23	Pelotas (Brazil)	179	22.27	6.38	0.76	-0.10
	Beijing (China)	433	23.05	6.70	0.49	0.37
	Nagpur (India)	327	22.42	6.37	0.57	-0.13
	Nairobi (Kenya)	233	22.49	6.44	0.76	-0.01
	Muscat (Oman)	209	22.11	6.31	0.71	-0.20
	Oxford (UK)	183	22.50	6.40	0.74	-0.07
	Turin (Italy)	168	21.78	6.27	0.72	-0.26
	**All Sites**	1732	22.48	6.45	0.67	0.00
24-26	Pelotas (Brazil)	209	25.49	7.63	0.67	0.12
	Beijing (China)	41	24.54	7.20	0.61	-0.52
	Nagpur (India)	303	25.56	7.41	0.70	-0.20
	Nairobi (Kenya)	199	25.41	7.46	0.74	-0.13
	Muscat (Oman)	330	25.45	7.53	0.59	-0.03
	Oxford (UK)	180	25.60	7.56	0.68	0.03
	Turin (Italy)	226	25.95	7.81	0.62	0.39
	**All Sites**	1488	25.54	7.55	0.67	0.00

GA (weeks)	Study site	Sample Total	Sample mean GA	OLD Adj. mean	OLD Adj. SD	Standardised Effect
18-20	Pelotas (Brazil)	130	19.73	6.48	1.01	0.33
	Beijing (China)	118	18.49	5.78	1.19	-0.24
	Nagpur (India)	186	19.46	5.94	1.37	-0.11
	Nairobi (Kenya)	134	19.48	5.72	1.48	-0.28
	Muscat (Oman)	219	19.89	6.23	1.09	0.13
	Oxford (UK)	95	19.30	5.96	1.27	-0.09
	Turin (Italy)	78	20.17	6.44	1.01	0.29
	**All Sites**	960	19.52	6.07	1.25	0.00
21-23	Pelotas (Brazil)	181	22.26	6.92	1.04	-0.11
	Beijing (China)	434	23.05	7.36	0.80	0.37
	Nagpur (India)	328	22.42	7.01	0.87	-0.02
	Nairobi (Kenya)	233	22.48	6.76	0.97	-0.29
	Muscat (Oman)	209	22.11	6.90	0.87	-0.14
	Oxford (UK)	186	22.50	6.90	0.95	-0.14

	Turin (Italy)	168	21.78	6.98	0.90	-0.05
	**All Sites**	1739	22.48	7.03	0.92	0.00
24-26	Pelotas (Brazil)	209	25.49	8.22	0.94	0.18
	Beijing (China)	41	24.54	7.91	0.70	-0.14
	Nagpur (India)	304	25.56	7.97	0.99	-0.07
	Nairobi (Kenya)	198	25.41	7.94	1.08	-0.10
	Muscat (Oman)	330	25.45	7.81	0.89	-0.24
	Oxford (UK)	180	25.60	8.07	0.97	0.03
	Turin (Italy)	225	25.95	8.40	0.98	0.36
	**All Sites**	1487	25.54	8.04	0.98	0.00

GA (weeks)	Study site	Sample Total	Sample mean GA	TLD Adj. mean	TLD Adj. SD	Standardised Effect
18-20	Pelotas (Brazil)	130	19.73	5.22	0.55	0.35
	Beijing (China)	117	18.50	4.81	0.64	-0.24
	Nagpur (India)	182	19.47	4.86	0.80	-0.17
	Nairobi (Kenya)	131	19.50	4.79	0.73	-0.27
	Muscat (Oman)	218	19.89	5.11	0.66	0.19
	Oxford (UK)	95	19.30	4.93	0.69	-0.07
	Turin (Italy)	78	20.17	5.10	0.65	0.17
	**All Sites**	951	19.53	4.98	0.70	0.00
21-23	Pelotas (Brazil)	180	22.26	5.71	0.81	-0.01
	Beijing (China)	436	23.05	5.99	0.67	0.38
	Nagpur (India)	329	22.41	5.61	0.71	-0.14
	Nairobi (Kenya)	233	22.50	5.60	0.81	-0.15
	Muscat (Oman)	209	22.11	5.57	0.65	-0.19
	Oxford (UK)	184	22.49	5.66	0.85	-0.08
	Turin (Italy)	168	21.78	5.60	0.62	-0.15
	**All Sites**	1739	22.48	5.71	0.75	0.00
24-26	Pelotas (Brazil)	209	25.49	6.90	0.75	0.26
	Beijing (China)	41	24.54	6.52	0.72	-0.20
	Nagpur (India)	304	25.56	6.62	0.82	-0.09
	Nairobi (Kenya)	199	25.41	6.56	0.90	-0.16
	Muscat (Oman)	330	25.45	6.48	0.76	-0.25
	Oxford (UK)	180	25.60	6.71	0.77	0.03
	Turin (Italy)	225	25.95	7.01	0.85	0.39
	**All Sites**	1488	25.54	6.69	0.83	0.00

GA (weeks)	Study site	Sample Total	Sample mean GA	ILD Adj. mean	ILD Adj. SD	Standardised Effect
18-20	Pelotas (Brazil)	130	19.73	7.06	0.72	0.33
	Beijing (China)	117	18.50	6.38	0.61	-0.57
	Nagpur (India)	185	19.46	6.68	0.84	-0.18
	Nairobi (Kenya)	131	19.50	6.78	0.73	-0.05
	Muscat (Oman)	219	19.89	6.99	0.69	0.22
	Oxford (UK)	96	19.30	6.66	0.77	-0.20
	Turin (Italy)	78	20.17	7.13	0.56	0.42
	**All Sites**	956	19.52	6.82	0.75	0.00
21-23	Pelotas (Brazil)	181	22.26	8.33	1.05	0.01
	Beijing (China)	434	23.05	8.60	0.88	0.29
	Nagpur (India)	330	22.41	8.14	0.93	-0.17
	Nairobi (Kenya)	235	22.49	8.41	1.11	0.10
	Muscat (Oman)	209	22.11	8.03	0.92	-0.29
	Oxford (UK)	184	22.49	8.39	0.93	0.07
	Turin (Italy)	168	21.78	8.05	1.02	-0.26
	**All Sites**	1741	22.48	8.31	0.98	0.00
24-26	Pelotas (Brazil)	209	25.49	10.66	1.08	0.13
	Beijing (China)	41	24.54	9.38	1.03	-0.88
	Nagpur (India)	304	25.56	10.39	1.27	-0.08
	Nairobi (Kenya)	199	25.41	10.31	1.41	-0.14
	Muscat (Oman)	329	25.45	10.27	1.20	-0.17
	Oxford (UK)	180	25.60	10.49	1.19	0.00
	Turin (Italy)	226	25.95	11.14	1.21	0.51
	**All Sites**	1488	25.54	10.49	1.27	0.00

GA (weeks)	Study site	Sample Total	Sample mean GA	FLT Adj. mean	FLT Adj. SD	Standardised Effect
18-20	Pelotas (Brazil)	129	19.72	2.27	0.14	-0.06
	Beijing (China)	116	18.49	2.33	0.23	0.27
	Nagpur (India)	184	19.46	2.32	0.19	0.24
	Nairobi (Kenya)	131	19.49	2.31	0.23	0.20
	Muscat (Oman)	218	19.89	2.23	0.12	-0.28
	Oxford (UK)	96	19.30	2.27	0.17	-0.05
	Turin (Italy)	78	20.17	2.21	0.10	-0.37
	**All Sites**	952	19.52	2.28	0.18	0.00
21-23	Pelotas (Brazil)	179	22.27	2.25	0.12	0.17
	Beijing (China)	433	23.05	2.22	0.10	-0.14

	Nagpur (India)	326	22.42	2.25	0.12	0.12
	Nairobi (Kenya)	232	22.50	2.24	0.11	0.06
	Muscat (Oman)	209	22.11	2.22	0.11	-0.14
	Oxford (UK)	188	22.50	2.24	0.12	0.07
	Turin (Italy)	168	21.78	2.23	0.11	-0.03
	**All Sites**	1735	22.48	2.23	0.11	0.00
24-26	Pelotas (Brazil)	209	25.49	2.29	0.10	0.16
	Beijing (China)	41	24.54	2.25	0.11	-0.25
	Nagpur (India)	303	25.56	2.27	0.09	-0.01
	Nairobi (Kenya)	200	25.42	2.28	0.11	0.09
	Muscat (Oman)	329	25.44	2.26	0.10	-0.19
	Oxford (UK)	180	25.60	2.28	0.10	0.07
	Turin (Italy)	226	25.95	2.28	0.10	0.04
	**All Sites**	1488	25.54	2.27	0.10	0.00

GA (weeks)	Study site	Sample Total	Sample mean GA	PLT Adj. mean	PLT Adj. SD	Standardised Effect
18-20	Pelotas (Brazil)	128	19.72	2.10	0.12	-0.12
	Beijing (China)	118	18.49	2.13	0.15	0.10
	Nagpur (India)	184	19.47	2.13	0.16	0.10
	Nairobi (Kenya)	131	19.48	2.14	0.18	0.17
	Muscat (Oman)	218	19.89	2.10	0.11	-0.13
	Oxford (UK)	94	19.30	2.12	0.16	0.01
	Turin (Italy)	78	20.17	2.09	0.11	-0.14
	**All Sites**	951	19.52	2.11	0.14	0.00
21-23	Pelotas (Brazil)	177	22.27	2.09	0.09	0.19
	Beijing (China)	434	23.05	2.06	0.07	-0.13
	Nagpur (India)	327	22.42	2.06	0.07	-0.16
	Nairobi (Kenya)	233	22.49	2.09	0.10	0.24
	Muscat (Oman)	208	22.12	2.06	0.08	-0.11
	Oxford (UK)	184	22.49	2.08	0.09	0.12
	Turin (Italy)	168	21.78	2.08	0.09	0.11
	**All Sites**	1731	22.48	2.07	0.08	0.00
24-26	Pelotas (Brazil)	209	25.49	2.18	0.11	0.08
	Beijing (China)	41	24.54	2.08	0.08	-0.81

	Nagpur (India)	304	25.56	2.16	0.10	-0.11
	Nairobi (Kenya)	199	25.42	2.19	0.12	0.21
	Muscat (Oman)	330	25.45	2.15	0.09	-0.22
	Oxford (UK)	180	25.60	2.18	0.11	0.08
	Turin (Italy)	228	25.95	2.20	0.10	0.30
	**All Sites**	1491	25.54	2.17	0.11	0.00

GA (weeks)	Study site	Sample Total	Sample mean GA	OLT Adj. mean	OLT Adj. SD	Standardised Effect
18-20	Pelotas (Brazil)	128	19.71	2.02	0.20	-0.16
	Beijing (China)	118	18.49	2.07	0.31	0.02
	Nagpur (India)	184	19.46	2.12	0.31	0.20
	Nairobi (Kenya)	129	19.49	2.12	0.31	0.21
	Muscat (Oman)	218	19.89	2.03	0.16	-0.13
	Oxford (UK)	95	19.30	2.05	0.27	-0.06
	Turin (Italy)	78	20.17	2.03	0.17	-0.15
	**All Sites**	950	19.52	2.06	0.26	0.00
21-23	Pelotas (Brazil)	180	22.26	2.08	0.13	0.03
	Beijing (China)	436	23.05	2.07	0.09	-0.04
	Nagpur (India)	324	22.42	2.07	0.10	-0.08
	Nairobi (Kenya)	233	22.49	2.11	0.12	0.35
	Muscat (Oman)	208	22.11	2.06	0.10	-0.11
	Oxford (UK)	186	22.50	2.08	0.12	0.04
	Turin (Italy)	166	21.78	2.06	0.11	-0.16
	**All Sites**	1733	22.48	2.08	0.11	0.00
24-26	Pelotas (Brazil)	209	25.49	2.15	0.09	0.01
	Beijing (China)	41	24.54	2.12	0.07	-0.30
	Nagpur (India)	304	25.56	2.14	0.09	-0.07
	Nairobi (Kenya)	200	25.42	2.16	0.10	0.10
	Muscat (Oman)	329	25.45	2.14	0.08	-0.09
	Oxford (UK)	180	25.60	2.15	0.09	-0.01
	Turin (Italy)	228	25.95	2.16	0.08	0.19
	**All Sites**	1491	25.54	2.15	0.09	0.00

GA (weeks)	Study site	Sample Total	Sample mean GA	TLT Adj. mean	TLT Adj. SD	Standardised Effect
18-20	Pelotas (Brazil)	129	19.72	2.21	0.11	-0.01
	Beijing (China)	117	18.50	2.10	0.10	-0.82
	Nagpur (India)	183	19.46	2.24	0.15	0.21

	Nairobi (Kenya)	134	19.48	2.23	0.15	0.14
	Muscat (Oman)	217	19.89	2.23	0.12	0.10
	Oxford (UK)	96	19.30	2.20	0.18	-0.08
	Turin (Italy)	78	20.17	2.26	0.14	0.32
	**All Sites**	954	19.52	2.21	0.14	0.00
21-23	Pelotas (Brazil)	181	22.26	2.37	0.12	-0.01
	Beijing (China)	436	23.05	2.39	0.12	0.21
	Nagpur (India)	329	22.41	2.36	0.11	-0.10
	Nairobi (Kenya)	237	22.49	2.37	0.11	0.01
	Muscat (Oman)	209	22.12	2.34	0.11	-0.25
	Oxford (UK)	182	22.49	2.40	0.13	0.21
	Turin (Italy)	168	21.78	2.34	0.13	-0.26
	**All Sites**	1742	22.48	2.37	0.12	0.00
24-26	Pelotas (Brazil)	209	25.49	2.51	0.10	0.19
	Beijing (China)	41	24.54	2.45	0.11	-0.42
	Nagpur (India)	304	25.56	2.50	0.10	0.04
	Nairobi (Kenya)	200	25.42	2.48	0.09	-0.09
	Muscat (Oman)	330	25.45	2.46	0.09	-0.31
	Oxford (UK)	179	25.61	2.51	0.10	0.14
	Turin (Italy)	228	25.95	2.52	0.08	0.27
	**All Sites**	1491	25.54	2.49	0.09	0.00

GA (weeks)	Study site	Sample Total	Sample mean GA	ILT Adj. mean	ILT Adj. SD	Standardised Effect
18-20	Pelotas (Brazil)	130	19.73	2.18	0.17	0.15
	Beijing (China)	118	18.49	2.00	0.11	-0.88
	Nagpur (India)	183	19.46	2.16	0.17	0.01
	Nairobi (Kenya)	130	19.51	2.16	0.19	0.02
	Muscat (Oman)	218	19.89	2.20	0.17	0.25
	Oxford (UK)	93	19.31	2.12	0.19	-0.20
	Turin (Italy)	78	20.17	2.26	0.16	0.60
	**All Sites**	950	19.52	2.16	0.18	0.00
21-23	Pelotas (Brazil)	180	22.27	2.58	0.23	-0.01
	Beijing (China)	433	23.05	2.61	0.20	0.14
	Nagpur (India)	330	22.41	2.57	0.21	-0.07
	Nairobi (Kenya)	237	22.49	2.63	0.25	0.23
	Muscat (Oman)	210	22.11	2.52	0.22	-0.27
	Oxford (UK)	182	22.47	2.62	0.24	0.18

	Turin (Italy)	167	21.78	2.50	0.22	-0.38
	**All Sites**	1739	22.47	2.58	0.22	0.00
24-26	Pelotas (Brazil)	208	25.49	3.01	0.26	0.02
	Beijing (China)	41	24.54	2.79	0.21	-0.84
	Nagpur (India)	304	25.56	3.02	0.26	0.06
	Nairobi (Kenya)	200	25.42	3.03	0.25	0.11
	Muscat (Oman)	330	25.45	2.95	0.24	-0.24
	Oxford (UK)	179	25.61	3.02	0.27	0.04
	Turin (Italy)	228	25.95	3.08	0.23	0.28
	**All Sites**	1490	25.54	3.01	0.25	0.00

Supplementary Table 6 - Standardised Sex differences (SSD) for each image derived phenotype (IDP). Number of samples excluding outliers lying >4SD above the mean at each complete gestational age (GA). The tables are separated by IDP: total brain volume (TBV), cortical plate volume (CoPV), white matter volume (WMV), deep grey matter volume (DGMV), cerebellum volume (CBV), thalamus volume (ThV), lateral ventricles volume (LVV), choroid plexus volume (ChPV), frontal horns volume (FHV), brainstem volume (BSV), cavum septum volume (CSPV), cortical plate suface area (CoPSA), Sylvian fissure depth (SFD), frontal lobe volume (FLV), temporal lobe volume (TLV), parietal lobe volume (PLV), occipital lobe volume (OLV), insular lobe volume (ILV), frontal lobe depth (FLD), temporal lobe depth (TLD), parietal lobe depth (PLD), occipital lobe depth (OLD), insular lobe depth (ILD), frontal lobe thickness (FLT), temporal lobe thickness (TLT), parietal lobe thickness (PLT), occipital lobe thickness (OLT) and insular lobe thickness (ILT).

GA (weeks)	Newborn Sex	Sample Total	Sample mean age	TBV Adj. mean	TBV Adj. SD	Standardised Effect
	Female	497	19.56	67.39	13.6	-0.17
18-20	Male	460	19.48	72.7	16.33	0.18
	Pooled	957	19.52	69.94	15.2	0
	Female	869	22.47	115.07	21.77	-0.18
21-23	Male	879	22.49	123.29	22.67	0.18
	Pooled	1748	22.48	119.21	22.6	0
24-26	Female	775	25.54	182.14	29.58	-0.24
	Male	716	25.54	198.04	33.39	0.25
	Pooled	1491	25.54	189.77	32.45	0
GA (weeks)	Newborn Sex	Sample Total	Sample mean age	CoPV Adj. mean	CoPV Adj. SD	Standardised Effect
18-20	Female	496	19.56	5.92	1.39	-0.13
	Male	453	19.49	6.33	1.64	0.14
	Pooled	949	19.52	6.12	1.53	0
21-23	Female	865	22.47	9.71	1.95	-0.14
	Male	875	22.49	10.28	2	0.14
	Pooled	1740	22.48	10	2	0
24-26	Female	775	25.54	14.89	2.26	-0.21
	Male	717	25.54	15.95	2.42	0.23
	Pooled	1492	25.54	15.4	2.39	0
GA (weeks)	Newborn Sex	Sample Total	Sample mean age	CoPSA Adj. mean	CoPSA Adj. SD	Standardised Effect
18-20	Female	495	19.56	66.77	10.09	-0.16
	Male	453	19.49	70.43	12.14	0.17
	Pooled	948	19.52	68.52	11.26	0
21-23	Female	867	22.47	100.09	15.23	-0.16
	Male	879	22.49	105.07	15.71	0.16
	Pooled	1746	22.48	102.6	15.67	0
24-26	Female	775	25.54	140.77	15.54	-0.21
	Male	717	25.54	147.86	16.41	0.23
	Pooled	1492	25.54	144.18	16.35	0
GA (weeks)	Newborn Sex	Sample Total	Sample mean age	WMV Adj. mean	WMV Adj. SD	Standardised Effect
18-20	Female	495	19.56	6.59	1.83	-0.15
	Male	453	19.49	7.26	2.3	0.17
	Pooled	948	19.52	6.91	2.1	0

21-23	Female	867	22.47	13.64	3.61	-0.15
	Male	879	22.49	14.76	3.84	0.15
	Pooled	1746	22.48	14.21	3.76	0
24-26	Female	775	25.54	24.1	4.43	-0.2
	Male	717	25.54	26.05	4.78	0.22
	Pooled	1492	25.54	25.04	4.7	0
GA (weeks)	Newborn Sex	Sample Total	Sample mean age	DGMV Adj. mean	DGMV Adj. SD	Standardised Effect
18-20	Female	495	19.56	3.23	0.63	-0.17
	Male	457	19.48	3.48	0.76	0.19
	Pooled	952	19.52	3.35	0.71	0
21-23	Female	868	22.47	5.45	1.07	-0.15
	Male	878	22.49	5.78	1.11	0.15
	Pooled	1746	22.48	5.62	1.1	0
24-26	Female	775	25.54	8.43	1.33	-0.19
	Male	716	25.54	9.01	1.48	0.21
	Pooled	1491	25.54	8.71	1.43	0
GA (weeks)	Newborn Sex	Sample Total	Sample mean age	CBV Adj. mean	CBV Adj. SD	Standardised Effect
18-20	Female	496	19.56	0.71	0.17	-0.14
	Male	454	19.49	0.76	0.2	0.15
	Pooled	950	19.52	0.73	0.19	0
21-23	Female	865	22.47	1.25	0.29	-0.17
	Male	876	22.49	1.36	0.31	0.16
	Pooled	1741	22.48	1.3	0.31	0
24-26	Female	775	25.54	2.03	0.38	-0.19
	Male	715	25.54	2.19	0.4	0.21
	Pooled	1490	25.54	2.1	0.4	0
GA (weeks)	Newborn Sex	Sample Total	Sample mean age	ChPV Adj. mean	ChPV Adj. SD	Standardised Effect
18-20	Female	499	19.56	0.71	0.14	-0.09
	Male	463	19.48	0.74	0.15	0.1
	Pooled	962	19.52	0.73	0.15	0
21-23	Female	871	22.47	0.76	0.17	-0.06
	Male	880	22.49	0.78	0.18	0.06
	Pooled	1751	22.48	0.77	0.18	0
24-26	Female	775	25.54	0.75	0.17	-0.03
	Male	715	25.54	0.76	0.17	0.03
	Pooled	1490	25.54	0.75	0.17	0
GA (weeks)	Newborn Sex	Sample Total	Sample mean age	LVV Adj. mean	LVV Adj. SD	Standardised Effect
18-20	Female	499	19.56	0.13	0.04	-0.14
	Male	462	19.48	0.14	0.04	0.15
	Pooled	961	19.52	0.14	0.04	0
21-23	Female	870	22.47	0.19	0.05	-0.12
	Male	880	22.49	0.2	0.05	0.12
	Pooled	1750	22.48	0.2	0.05	0

24-26	Female	775	25.54	0.24	0.06	-0.15
	Male	717	25.54	0.26	0.06	0.17
	Pooled	1492	25.54	0.25	0.06	0
GA (weeks)	Newborn Sex	Sample Total	Sample mean age	FHV Adj. mean	FHV Adj. SD	Standardised Effect
18-20	Female	498	19.56	0.18	0.02	-0.24
	Male	462	19.48	0.19	0.03	0.26
	Pooled	960	19.52	0.19	0.03	0
21-23	Female	870	22.47	0.24	0.04	-0.16
	Male	879	22.49	0.26	0.04	0.16
	Pooled	1749	22.48	0.25	0.04	0
24-26	Female	775	25.54	0.33	0.05	-0.19
	Male	717	25.54	0.35	0.05	0.2
	Pooled	1492	25.54	0.34	0.05	0
GA (weeks)	Newborn Sex	Sample Total	Sample mean age	BSV Adj. mean	BSVAdj. SD	Standardised Effect
18-20	Female	498	19.56	0.41	0.06	-0.14
	Male	459	19.48	0.43	0.07	0.15
	Pooled	957	19.52	0.42	0.07	0
21-23	Female	868	22.47	0.57	0.08	-0.13
	Male	878	22.49	0.59	0.08	0.13
	Pooled	1746	22.48	0.58	0.08	0
24-26	Female	774	25.55	0.79	0.12	-0.2
	Male	716	25.54	0.85	0.13	0.21
	Pooled	1490	25.54	0.82	0.13	0
GA (weeks)	Newborn Sex	Sample Total	Sample mean age	ThV Adj. mean	ThV Adj. SD	Standardised Effect
18-20	Female	499	19.56	0.45	0.09	-0.16
	Male	460	19.48	0.48	0.1	0.17
	Pooled	959	19.52	0.46	0.1	0
21-23	Female	869	22.47	0.7	0.12	-0.11
	Male	878	22.49	0.73	0.13	0.11
	Pooled	1747	22.48	0.71	0.13	0
24-26	Female	774	25.54	1.02	0.16	-0.16
	Male	717	25.54	1.07	0.17	0.17
	Pooled	1491	25.54	1.05	0.16	0
GA (weeks)	Newborn Sex	Sample Total	Sample mean age	CSPV Adj. mean	CSPV Adj. SD	Standardised Effect
18-20	Female	496	19.56	0.13	0.04	-0.15
	Male	459	19.48	0.14	0.05	0.16
	Pooled	955	19.52	0.13	0.04	0
21-23	Female	868	22.47	0.28	0.08	-0.15
	Male	878	22.49	0.3	0.08	0.15
	Pooled	1746	22.48	0.29	0.08	0
24-26	Female	775	25.54	0.48	0.1	-0.2
	Male	716	25.54	0.53	0.11	0.21
	Pooled	1491	25.54	0.5	0.11	0

GA (weeks)	Newborn Sex	Sample Total	Sample mean age	SFD Adj. mean	SFD Adj. SD	Standardised Effect
18-20	Female	497	19.56	6.82	0.74	-0.08
	Male	459	19.49	6.95	0.83	0.09
	Pooled	956	19.52	6.88	0.79	0
21-23	Female	866	22.47	8.28	1	-0.14
	Male	873	22.48	8.57	1.02	0.14
	Pooled	1739	22.48	8.43	1.02	0
24-26	Female	773	25.54	10.36	1.22	-0.15
	Male	715	25.54	10.77	1.33	0.16
	Pooled	1488	25.54	10.56	1.29	0
GA (weeks)	Newborn Sex	Sample Total	Sample mean age	FLV Adj. mean	FLV Adj. SD	Standardised Effect
18-20	Female	496	19.56	2.15	0.44	-0.14
	Male	454	19.49	2.28	0.52	0.15
	Pooled	950	19.53	2.21	0.48	0
21-23	Female	865	22.47	3.42	0.73	-0.14
	Male	872	22.49	3.63	0.74	0.14
	Pooled	1737	22.48	3.52	0.74	0
24-26	Female	775	25.54	5.28	0.87	-0.23
	Male	717	25.54	5.71	0.94	0.24
	Pooled	1492	25.54	5.49	0.93	0
GA (weeks)	Newborn Sex	Sample Total	Sample mean age	PLV Adj. mean	PLV Adj. SD	Standardised Effect
18-20	Female	495	19.56	1.63	0.36	-0.11
	Male	452	19.48	1.73	0.43	0.13
	Pooled	947	19.52	1.68	0.4	0
21-23	Female	864	22.47	2.38	0.41	-0.11
	Male	873	22.49	2.47	0.41	0.11
	Pooled	1737	22.48	2.42	0.41	0
24-26	Female	775	25.54	3.37	0.57	-0.19
	Male	716	25.54	3.61	0.61	0.21
	Pooled	1491	25.54	3.49	0.6	0
GA (weeks)	Newborn Sex	Sample Total	Sample mean age	OLV Adj. mean	OLV Adj. SD	Standardised Effect
18-20	Female	495	19.56	0.93	0.32	-0.1
	Male	451	19.48	1	0.36	0.11
	Pooled	946	19.52	0.97	0.34	0
21-23	Female	867	22.47	1.65	0.42	-0.1
	Male	875	22.49	1.73	0.42	0.1
	Pooled	1742	22.48	1.69	0.42	0
24-26	Female	775	25.54	2.58	0.42	-0.17
	Male	716	25.54	2.73	0.46	0.18
	Pooled	1491	25.54	2.65	0.44	0
GA (weeks)	Newborn Sex	Sample Total	Sample mean age	TLV Adj. mean	TLV Adj. SD	Standardised Effect
18-20	Female	496	19.56	1.03	0.32	-0.11
	Male	453	19.49	1.11	0.37	0.13

	Pooled	949	19.53	1.06	0.34	0
21-23	Female	866	22.47	1.91	0.48	-0.15
	Male	876	22.49	2.06	0.49	0.15
	Pooled	1742	22.48	1.98	0.49	0
24-26	Female	775	25.54	3.06	0.51	-0.16
	Male	716	25.54	3.24	0.53	0.18
	Pooled	1491	25.54	3.15	0.53	0
GA (weeks)	Newborn Sex	Sample Total	Sample mean age	ILV Adj. mean	ILV Adj. SD	Standardised Effect
18-20	Female	495	19.56	0.13	0.04	-0.15
	Male	455	19.48	0.15	0.05	0.17
	Pooled	950	19.52	0.14	0.05	0
21-23	Female	867	22.47	0.29	0.09	-0.15
	Male	876	22.49	0.32	0.09	0.15
	Pooled	1743	22.48	0.31	0.09	0
24-26	Female	775	25.54	0.51	0.11	-0.16
	Male	715	25.55	0.55	0.12	0.17
	Pooled	1490	25.54	0.53	0.12	0
GA (weeks)	Newborn Sex	Sample Total	Sample mean age	FLD Adj. mean	FLD Adj. SD	Standardised Effect
18-20	Female	497	19.56	5.16	0.82	-0.03
	Male	462	19.48	5.22	0.96	0.03
	Pooled	959	19.52	5.19	0.89	0
21-23	Female	863	22.47	5.67	0.58	-0.13
	Male	871	22.49	5.82	0.59	0.13
	Pooled	1734	22.48	5.75	0.59	0
24-26	Female	772	25.54	6.06	0.68	-0.16
	Male	715	25.55	6.3	0.74	0.17
	Pooled	1487	25.54	6.18	0.72	0
GA (weeks)	Newborn Sex	Sample Total	Sample mean age	PLD Adj. mean	PLD Adj. SD	Standardised Effect
18-20	Female	493	19.56	5.31	0.85	-0.03
	Male	458	19.48	5.37	0.87	0.04
	Pooled	951	19.52	5.34	0.86	0
21-23	Female	862	22.47	6.38	0.66	-0.1
	Male	870	22.49	6.52	0.68	0.1
	Pooled	1732	22.48	6.45	0.67	0
24-26	Female	774	25.54	7.41	0.61	-0.2
	Male	714	25.54	7.69	0.72	0.21
	Pooled	1488	25.54	7.55	0.67	0
GA (weeks)	Newborn Sex	Sample Total	Sample mean age	OLD Adj. mean	OLD Adj. SD	Standardised Effect
18-20	Female	498	19.56	6.03	1.22	-0.04
	Male	462	19.48	6.12	1.28	0.04
	Pooled	960	19.52	6.07	1.25	0
21-23	Female	865	22.47	6.95	0.94	-0.08
	Male	874	22.49	7.1	0.9	0.08

	Pooled	1739	22.48	7.03	0.92	0
24-26	Female	772	25.54	7.86	0.87	-0.18
	Male	715	25.54	8.24	1.05	0.2
	Pooled	1487	25.54	8.04	0.98	0
GA (weeks)	Newborn Sex	Sample Total	Sample mean age	TLD Adj. mean	TLD Adj. SD	Standardised Effect
18-20	Female	495	19.56	4.96	0.7	-0.03
	Male	456	19.49	5	0.71	0.03
	Pooled	951	19.53	4.98	0.7	0
21-23	Female	865	22.47	5.65	0.73	-0.09
	Male	874	22.49	5.78	0.75	0.09
	Pooled	1739	22.48	5.71	0.75	0
24-26	Female	774	25.54	6.56	0.78	-0.15
	Male	714	25.54	6.82	0.85	0.16
	Pooled	1488	25.54	6.69	0.83	0
GA (weeks)	Newborn Sex	Sample Total	Sample mean age	ILD Adj. mean	ILD Adj. SD	Standardised Effect
18-20	Female	498	19.56	6.75	0.72	-0.09
	Male	458	19.49	6.89	0.78	0.1
	Pooled	956	19.52	6.82	0.75	0
21-23	Female	867	22.46	8.17	0.97	-0.15
	Male	874	22.49	8.46	0.98	0.15
	Pooled	1741	22.48	8.31	0.98	0
24-26	Female	773	25.54	10.29	1.21	-0.15
	Male	715	25.54	10.7	1.3	0.17
	Pooled	1488	25.54	10.49	1.27	0
GA (weeks)	Newborn Sex	Sample Total	Sample mean age	FLT Adj. mean	FLT Adj. SD	Standardised Effect
18-20	Female	495	19.56	2.27	0.17	-0.03
	Male	457	19.49	2.28	0.19	0.03
	Pooled	952	19.52	2.28	0.18	0
21-23	Female	863	22.47	2.23	0.11	-0.02
	Male	872	22.49	2.24	0.11	0.02
	Pooled	1735	22.48	2.23	0.11	0
24-26	Female	772	25.54	2.26	0.1	-0.11
	Male	716	25.54	2.29	0.1	0.12
	Pooled	1488	25.54	2.27	0.1	0
GA (weeks)	Newborn Sex	Sample Total	Sample mean age	PLT Adj. mean	PLT Adj. SD	Standardised Effect
18-20	Female	492	19.56	2.11	0.13	-0.02
	Male	459	19.48	2.12	0.15	0.02
	Pooled	951	19.52	2.11	0.14	0
21-23	Female	859	22.47	2.07	0.08	-0.02
	Male	872	22.49	2.07	0.08	0.02
	Pooled	1731	22.48	2.07	0.08	0
24-26	Female	775	25.54	2.16	0.1	-0.13
	Male	716	25.54	2.18	0.1	0.14

	Pooled	1491	25.54	2.17	0.11	0
GA (weeks)	Newborn Sex	Sample Total	Sample mean age	OLT Adj. mean	OLT Adj. SD	Standardised Effect
18-20	Female	494	19.56	2.06	0.25	-0.02
	Male	456	19.48	2.07	0.26	0.02
	Pooled	950	19.52	2.06	0.26	0
21-23	Female	861	22.47	2.07	0.11	-0.03
	Male	872	22.49	2.08	0.1	0.03
	Pooled	1733	22.48	2.08	0.11	0
24-26	Female	774	25.54	2.14	0.08	-0.11
	Male	717	25.54	2.16	0.09	0.11
	Pooled	1491	25.54	2.15	0.09	0
GA (weeks)	Newborn Sex	Sample Total	Sample mean age	TLT Adj. mean	TLT Adj. SD	Standardised Effect
18-20	Female	495	19.56	2.21	0.14	-0.05
	Male	459	19.48	2.22	0.15	0.05
	Pooled	954	19.52	2.21	0.14	0
21-23	Female	870	22.47	2.36	0.12	-0.1
	Male	872	22.49	2.38	0.12	0.1
	Pooled	1742	22.48	2.37	0.12	0
24-26	Female	775	25.54	2.48	0.09	-0.12
	Male	716	25.54	2.5	0.09	0.13
	Pooled	1491	25.54	2.49	0.09	0
GA (weeks)	Newborn Sex	Sample Total	Sample mean age	ILT Adj. mean	ILT Adj. SD	Standardised Effect
18-20	Female	494	19.56	2.13	0.17	-0.14
	Male	456	19.48	2.18	0.19	0.15
	Pooled	950	19.52	2.16	0.18	0
21-23	Female	867	22.47	2.56	0.22	-0.11
	Male	872	22.48	2.61	0.22	0.11
	Pooled	1739	22.47	2.58	0.22	0
24-26	Female	775	25.54	2.98	0.25	-0.12
	Male	715	25.55	3.04	0.25	0.13
	Pooled	1490	25.54	3.01	0.25	0

Supplementary Table 7 - Standardised differences between cerebral hemispheres for each image derived phenotype (IDP). Number of samples excluding outliers lying >4SD above the mean at each complete gestational age. The tables are separated by IDP: total brain volume (TBV), cortical plate volume (CoPV), white matter volume (WMV), deep grey matter volume (DGMV), cerebellum

volume (CBV), thalamus volume (ThV), lateral ventricles volume (LVV), choroid plexus volume (ChPV), frontal horns volume (FHV), brainstem volume (BSV), cavum septum volume (CSPV), cortical plate suface area (CoPSA), Sylvian fissure depth (SFD), frontal lobe volume (FLV), temporal lobe volume (TLV), parietal lobe volume (PLV), occipital lobe volume (OLV), insular lobe volume (ILV), frontal lobe depth (FLD), temporal lobe depth (TLD), parietal lobe depth (PLD), occipital lobe depth (OLD), insular lobe depth (ILD), frontal lobe thickness (FLT), temporal lobe thickness (TLT), parietal lobe thickness (PLT), occipital lobe thickness (OLT) and insular lobe thickness (ILT).

GA (weeks)	Cerebral Hemisphere	Sample Total	Sample mean age	CoPV Adj. mean	CoPV Adj. SD	Standardised Effect
18-20	left	469	19.45	68.94	14.17	-0.07
	right	488	19.59	70.91	16.08	0.06
	pooled	957	19.52	69.94	15.2	0
21-23	left	833	22.46	117.82	21.59	-0.06
	right	915	22.49	120.47	23.42	0.06
	pooled	1748	22.48	119.21	22.6	0
24-26	left	707	25.53	189.56	33.41	-0.01
	right	784	25.55	189.97	31.58	0.01
	pooled	1491	25.54	189.77	32.45	0
GA (weeks)	Cerebral Hemisphere	Sample Total	Sample mean age	CoPV Adj. mean	CoPV Adj. SD	Standardised Effect
18-20	left	466	19.45	5.94	1.44	-0.11
	right	483	19.59	6.28	1.6	0.11
	pooled	949	19.52	6.12	1.53	0
21-23	left	829	22.46	9.9	1.99	-0.05
	right	911	22.49	10.09	2	0.04
	pooled	1740	22.48	10	2	0
24-26	left	708	25.53	15.53	2.49	0.06
	right	784	25.55	15.28	2.29	-0.05
	pooled	1492	25.54	15.4	2.39	0
GA (weeks)	Cerebral Hemisphere	Sample Total	Sample mean age	CoPSA Adj. mean	CoPSA Adj. SD	Standardised Effect
18-20	left	465	19.45	66.73	10.35	-0.16
	right	483	19.59	70.25	11.83	0.15
	pooled	948	19.52	68.52	11.26	0
21-23	left	831	22.46	100.31	14.86	-0.15
	right	915	22.49	104.68	16.09	0.13
	pooled	1746	22.48	102.6	15.67	0
24-26	left	708	25.53	142.78	17.29	-0.09
	right	784	25.55	145.43	15.35	0.08
	pooled	1492	25.54	144.18	16.35	0
GA (weeks)	Cerebral Hemisphere	Sample Total	Sample mean age	WMV Adj. mean	WMV Adj. SD	Standardised Effect
18-20	left	465	19.45	6.44	1.79	-0.23
	right	483	19.59	7.36	2.26	0.22
	pooled	948	19.52	6.91	2.1	0
21-23	left	831	22.46	13.37	3.37	-0.22
	right	915	22.49	14.97	3.94	0.2
	pooled	1746	22.48	14.21	3.76	0

24-26	left	708	25.53	24.13	4.75	-0.19
	right	784	25.55	25.86	4.5	0.18
	pooled	1492	25.54	25.04	4.7	0
GA (weeks)	Cerebral Hemisphere	Sample Total	Sample mean age	DGMV Adj. mean	DGMV Adj. SD	Standardised Effect
18-20	left	468	19.45	3.23	0.65	-0.18
	right	484	19.59	3.48	0.74	0.17
	pooled	952	19.52	3.35	0.71	0
21-23	left	832	22.46	5.43	1.05	-0.17
	right	914	22.49	5.79	1.12	0.15
	pooled	1746	22.48	5.62	1.1	0
24-26	left	707	25.53	8.64	1.55	-0.05
	right	784	25.55	8.77	1.32	0.04
	pooled	1491	25.54	8.71	1.43	0
GA (weeks)	Cerebral Hemisphere	Sample Total	Sample mean age	CBV Adj. mean	CBV Adj. SD	Standardised Effect
18-20	left	467	19.45	0.72	0.18	-0.09
	right	483	19.59	0.75	0.2	0.08
	pooled	950	19.52	0.73	0.19	0
21-23	left	830	22.46	1.28	0.31	-0.09
	right	911	22.49	1.33	0.31	0.08
	pooled	1741	22.48	1.3	0.31	0
24-26	left	708	25.53	2.08	0.4	-0.05
	right	782	25.55	2.12	0.39	0.05
	pooled	1490	25.54	2.1	0.4	0
GA (weeks)	Cerebral Hemisphere	Sample Total	Sample mean age	ChPV Adj. mean	ChPV Adj. SD	Standardised Effect
18-20	left	473	19.45	0.76	0.14	0.21
	right	489	19.59	0.7	0.14	-0.21
	pooled	962	19.52	0.73	0.15	0
21-23	left	833	22.46	0.81	0.17	0.22
	right	918	22.49	0.74	0.17	-0.2
	pooled	1751	22.48	0.77	0.18	0
24-26	left	706	25.53	0.82	0.17	0.42
	right	784	25.55	0.69	0.14	-0.38
	pooled	1490	25.54	0.75	0.17	0
GA (weeks)	Cerebral Hemisphere	Sample Total	Sample mean age	LVV Adj. mean	LVV Adj. SD	Standardised Effect
18-20	left	472	19.45	0.16	0.04	0.46
	right	489	19.59	0.12	0.04	-0.44
	pooled	961	19.52	0.14	0.04	0
21-23	left	832	22.46	0.21	0.05	0.34
	right	918	22.49	0.18	0.05	-0.31
	pooled	1750	22.48	0.2	0.05	0
24-26	left	708	25.53	0.28	0.06	0.45
	right	784	25.55	0.22	0.05	-0.4
	pooled	1492	25.54	0.25	0.06	0

GA (weeks)	Cerebral Hemisphere	Sample Total	Sample mean age	FHV Adj. mean	FHV Adj. SD	Standardised Effect
18-20	left	472	19.45	0.19	0.02	0.13
	right	488	19.59	0.18	0.03	-0.13
	pooled	960	19.52	0.19	0.03	0
21-23	left	833	22.46	0.24	0.03	-0.27
	right	916	22.49	0.26	0.04	0.24
	pooled	1749	22.48	0.25	0.04	0
24-26	left	708	25.53	0.33	0.05	-0.26
	right	784	25.55	0.35	0.05	0.23
	pooled	1492	25.54	0.34	0.05	0
GA (weeks)	Cerebral Hemisphere	Sample Total	Sample mean age	BSV Adj. mean	BSV Adj. SD	Standardised Effect
18-20	left	472	19.45	0.41	0.06	-0.05
	right	485	19.59	0.42	0.07	0.05
	pooled	957	19.52	0.42	0.07	0
21-23	left	831	22.46	0.57	0.08	-0.1
	right	915	22.49	0.59	0.09	0.09
	pooled	1746	22.48	0.58	0.08	0
24-26	left	706	25.54	0.82	0.13	0
	right	784	25.55	0.82	0.12	0
	pooled	1490	25.54	0.82	0.13	0
GA (weeks)	Cerebral Hemisphere	Sample Total	Sample mean age	ThV Adj. mean	ThV Adj. SD	Standardised Effect
18-20	left	471	19.45	0.47	0.09	0.07
	right	488	19.59	0.46	0.1	-0.07
	pooled	959	19.52	0.46	0.1	0
21-23	left	832	22.46	0.71	0.12	-0.05
	right	915	22.49	0.72	0.13	0.05
	pooled	1747	22.48	0.71	0.13	0
24-26	left	707	25.53	1.05	0.17	0.01
	right	784	25.55	1.04	0.16	-0.01
	pooled	1491	25.54	1.05	0.16	0
GA (weeks)	Cerebral Hemisphere	Sample Total	Sample mean age	CSPV Adj. mean	CSPV Adj. SD	Standardised Effect
18-20	left	468	19.45	0.13	0.04	-0.07
	right	487	19.59	0.14	0.05	0.06
	pooled	955	19.52	0.13	0.04	0
21-23	left	831	22.46	0.28	0.08	-0.06
	right	915	22.49	0.29	0.08	0.06
	pooled	1746	22.48	0.29	0.08	0
24-26	left	708	25.53	0.5	0.11	-0.04
	right	783	25.55	0.51	0.1	0.03
	pooled	1491	25.54	0.5	0.11	0
GA (weeks)	Cerebral Hemisphere	Sample Total	Sample mean age	SFD Adj. mean	SFD Adj. SD	Standardised Effect
18-20	left	468	19.45	6.74	0.75	-0.18
	right	488	19.59	7.02	0.8	0.18

	pooled	956	19.52	6.88	0.79	0
21-23	left	830	22.46	8.32	1	-0.1
	right	909	22.49	8.52	1.03	0.09
	pooled	1739	22.48	8.43	1.02	0
24-26	left	708	25.53	10.53	1.19	-0.02
	right	780	25.55	10.58	1.38	0.02
	pooled	1488	25.54	10.56	1.29	0
GA (weeks)	Cerebral Hemisphere	Sample Total	Sample mean age	FLV Adj. mean	FLV Adj. SD	Standardised Effect
18-20	left	467	19.46	2.12	0.41	-0.19
	right	483	19.59	2.3	0.53	0.18
	pooled	950	19.53	2.21	0.48	0
21-23	left	831	22.46	3.44	0.7	-0.11
	right	906	22.49	3.6	0.77	0.1
	pooled	1737	22.48	3.52	0.74	0
24-26	left	708	25.53	5.52	1	0.04
	right	784	25.55	5.45	0.86	-0.04
	pooled	1492	25.54	5.49	0.93	0
GA (weeks)	Cerebral Hemisphere	Sample Total	Sample mean age	PLV Adj. mean	PLV Adj. SD	Standardised Effect
18-20	left	466	19.45	1.66	0.36	-0.05
	right	481	19.59	1.7	0.42	0.05
	pooled	947	19.52	1.68	0.4	0
21-23	left	825	22.46	2.45	0.4	0.06
	right	912	22.49	2.4	0.42	-0.05
	pooled	1737	22.48	2.42	0.41	0
24-26	left	707	25.54	3.6	0.62	0.19
	right	784	25.55	3.38	0.57	-0.17
	pooled	1491	25.54	3.49	0.6	0
GA (weeks)	Cerebral Hemisphere	Sample Total	Sample mean age	OLV Adj. mean	OLV Adj. SD	Standardised Effect
18-20	left	464	19.46	0.93	0.34	-0.1
	right	482	19.59	1	0.35	0.09
	pooled	946	19.52	0.97	0.34	0
21-23	left	827	22.46	1.7	0.44	0.02
	right	915	22.49	1.68	0.4	-0.02
	pooled	1742	22.48	1.69	0.42	0
24-26	left	707	25.53	2.71	0.46	0.12
	right	784	25.55	2.61	0.43	-0.11
	pooled	1491	25.54	2.65	0.44	0
GA (weeks)	Cerebral Hemisphere	Sample Total	Sample mean age	TLV Adj. mean	TLV Adj. SD	Standardised Effect
18-20	left	466	19.46	1.04	0.33	-0.08
	right	483	19.59	1.09	0.35	0.08
	pooled	949	19.53	1.06	0.34	0
21-23	left	830	22.46	1.91	0.47	-0.16
	right	912	22.49	2.05	0.49	0.14
	pooled	1742	22.48	1.98	0.49	0

24-26	left	708	25.53	3.01	0.49	-0.26
	right	783	25.55	3.27	0.53	0.24
	pooled	1491	25.54	3.15	0.53	0
GA (weeks)	Cerebral Hemisphere	Sample Total	Sample mean age	ILV Adj. mean	ILV Adj. SD	Standardised Effect
18-20	left	466	19.45	0.13	0.05	-0.08
	right	484	19.59	0.14	0.05	0.08
	pooled	950	19.52	0.14	0.05	0
21-23	left	828	22.46	0.32	0.09	0.2
	right	915	22.5	0.29	0.08	-0.18
	pooled	1743	22.48	0.31	0.09	0
24-26	left	706	25.54	0.58	0.11	0.46
	right	784	25.55	0.48	0.09	-0.42
	pooled	1490	25.54	0.53	0.12	0
GA (weeks)	Cerebral Hemisphere	Sample Total	Sample mean age	FLD Adj. mean	FLD Adj. SD	Standardised Effect
18-20	left	472	19.45	5.27	0.83	0.09
	right	487	19.59	5.11	0.94	-0.09
	pooled	959	19.52	5.19	0.89	0
21-23	left	828	22.46	5.8	0.58	0.08
	right	906	22.49	5.7	0.6	-0.08
	pooled	1734	22.48	5.75	0.59	0
24-26	left	706	25.54	6.14	0.72	-0.05
	right	781	25.55	6.21	0.73	0.05
	pooled	1487	25.54	6.18	0.72	0
GA (weeks)	Cerebral Hemisphere	Sample Total	Sample mean age	PLD Adj. mean	PLD Adj. SD	Standardised Effect
18-20	left	469	19.45	5.4	0.81	0.06
	right	482	19.59	5.29	0.9	-0.06
	pooled	951	19.52	5.34	0.86	0
21-23	left	824	22.47	6.51	0.63	0.08
	right	908	22.49	6.4	0.7	-0.08
	pooled	1732	22.48	6.45	0.67	0
24-26	left	706	25.53	7.61	0.67	0.09
	right	782	25.55	7.49	0.67	-0.08
	pooled	1488	25.54	7.55	0.67	0
GA (weeks)	Cerebral Hemisphere	Sample Total	Sample mean age	OLD Adj. mean	OLD Adj. SD	Standardised Effect
18-20	left	471	19.45	6.44	1.19	0.29
	right	489	19.59	5.72	1.21	-0.28
	pooled	960	19.52	6.07	1.25	0
21-23	left	827	22.46	7.24	0.92	0.24
	right	912	22.49	6.83	0.88	-0.21
	pooled	1739	22.48	7.03	0.92	0
24-26	left	705	25.53	8.28	0.95	0.24
	right	782	25.55	7.83	0.96	-0.22
	pooled	1487	25.54	8.04	0.98	0

GA (weeks)	Cerebral Hemisphere	Sample Total	Sample mean age	TLD Adj. mean	TLD Adj. SD	Standardised Effect
18-20	left	465	19.46	4.81	0.69	-0.23
	right	486	19.59	5.14	0.67	0.22
	pooled	951	19.53	4.98	0.7	0
21-23	left	825	22.46	5.55	0.75	-0.22
	right	914	22.49	5.86	0.71	0.2
	pooled	1739	22.48	5.71	0.75	0
24-26	left	705	25.53	6.46	0.74	-0.28
	right	783	25.55	6.89	0.84	0.25
	pooled	1488	25.54	6.69	0.83	0
GA (weeks)	Cerebral Hemisphere	Sample Total	Sample mean age	ILD Adj. mean	ILD Adj. SD	Standardised Effect
18-20	left	468	19.46	6.71	0.71	-0.15
	right	488	19.59	6.92	0.78	0.14
	pooled	956	19.52	6.82	0.75	0
21-23	left	829	22.46	8.2	0.96	-0.11
	right	912	22.49	8.42	1	0.1
	pooled	1741	22.48	8.31	0.98	0
24-26	left	708	25.53	10.46	1.21	-0.02
	right	780	25.55	10.51	1.32	0.02
	pooled	1488	25.54	10.49	1.27	0
GA (weeks)	Cerebral Hemisphere	Sample Total	Sample mean age	FLT Adj. mean	FLT Adj. SD	Standardised Effect
18-20	left	471	19.45	2.27	0.14	-0.03
	right	481	19.59	2.28	0.21	0.03
	pooled	952	19.52	2.28	0.18	0
21-23	left	825	22.46	2.27	0.09	0.3
	right	910	22.5	2.2	0.12	-0.27
	pooled	1735	22.48	2.23	0.11	0
24-26	left	705	25.53	2.3	0.1	0.28
	right	783	25.55	2.25	0.09	-0.25
	pooled	1488	25.54	2.27	0.1	0
GA (weeks)	Cerebral Hemisphere	Sample Total	Sample mean age	PLT Adj. mean	PLT Adj. SD	Standardised Effect
18-20	left	471	19.45	2.09	0.1	-0.2
	right	480	19.59	2.14	0.17	0.2
	pooled	951	19.52	2.11	0.14	0
21-23	left	825	22.46	2.06	0.08	-0.2
	right	906	22.49	2.09	0.09	0.18
	pooled	1731	22.48	2.07	0.08	0
24-26	left	708	25.53	2.19	0.11	0.24
	right	783	25.55	2.15	0.09	-0.21
	pooled	1491	25.54	2.17	0.11	0
GA (weeks)	Cerebral Hemisphere	Sample Total	Sample mean age	OLT Adj. mean	OLT Adj. SD	Standardised Effect
18-20	left	471	19.45	2.03	0.23	-0.15
	right	479	19.59	2.1	0.27	0.14

	pooled	950	19.52	2.06	0.26	0
21-23	left	827	22.46	2.09	0.11	0.13
	right	906	22.5	2.06	0.11	-0.12
	pooled	1733	22.48	2.08	0.11	0
24-26	left	707	25.54	2.19	0.08	0.53
	right	784	25.55	2.11	0.07	-0.48
	pooled	1491	25.54	2.15	0.09	0
GA (weeks)	Cerebral Hemisphere	Sample Total	Sample mean age	TLT Adj. mean	TLT Adj. SD	Standardised Effect
18-20	left	469	19.45	2.23	0.14	0.12
	right	485	19.58	2.2	0.15	-0.11
	pooled	954	19.52	2.21	0.14	0
21-23	left	828	22.46	2.36	0.12	-0.08
	right	914	22.49	2.38	0.12	0.07
	pooled	1742	22.48	2.37	0.12	0
24-26	left	707	25.54	2.48	0.09	-0.12
	right	784	25.55	2.5	0.1	0.11
	pooled	1491	25.54	2.49	0.09	0
GA (weeks)	Cerebral Hemisphere	Sample Total	Sample mean age	ILT Adj. mean	ILT Adj. SD	Standardised Effect
18-20	left	466	19.46	2.16	0.19	0.03
	right	484	19.59	2.15	0.18	-0.03
	pooled	950	19.52	2.16	0.18	0
21-23	left	825	22.45	2.62	0.22	0.16
	right	914	22.49	2.55	0.22	-0.15
	pooled	1739	22.47	2.58	0.22	0
24-26	left	706	25.54	3.08	0.26	0.29
	right	784	25.55	2.94	0.23	-0.26
	pooled	1490	25.54	3.01	0.25	0

Supplementary Table 8: Formulae for fetal brain growth trajectories. Equations for estimating the mean and SD of each of the 28 image-derived phenotypes (IDPs) according to exact gestational age (GA) in weeks. The IDPs included are: total brain volume (TBV), cortical plate volume (CoPV), white matter volume (WMV), deep grey matter volume (DGMV), cerebellum volume (CBV), thalamus volume (ThV), lateral posterior ventricle horns volume (LVV), choroid plexus volume (ChPV), frontal horns volume (FHV), brainstem volume (BSV), cavum septum volume (CSPV), cortical plate surface area (CoPSA), Sylvian fissure depth (SFD), frontal lobe volume (FLV), temporal lobe volume (TLV), parietal lobe volume (PLV), occipital lobe volume (OLV), insular lobe volume (ILV), frontal lobe depth (FLD), temporal lobe depth (TLD), parietal lobe depth (PLD), occipital lobe depth (OLD), insular lobe depth (ILD), frontal lobe thickness (FLT), temporal lobe thickness (TLT), parietal lobe thickness (PLT), occipital lobe thickness (OLT), insular lobe thickness (ILT), relative ILV (rILV), relative PLV (rPLV) and their ratio. The IDPs are in cm^3 for all structures, except CoPA which is in cm^2 and the cortical thickness (LT), depth (LD) and SFD are all in mm. Number of samples excluding outliers lying >4SD above the mean at each complete gestational age. For SD, the reported equation is on the log scale and must be exponentiated to obtain the standard deviation.

	IDP	Number included scans		Regression Equation
Structure volume (cm^3)	**TBV**	4196	Mean	$1.954510 + 0.018205xGA^{3} + -0.178633xGA^{2}$
		4196	SD	$-0.702623 + 0.150265xGA$
	**CoPV**	4181	Mean	$213.602100 + -1055185.000000xGA^{-3} + 179286.700000xGA^{-2} -10465.890000xGA^{-1}$
		4181	SD	$-3.319410 + 0.276768xGA + -0.004899xGA^{2}$
	**WMV**	4186	Mean	$147.933791 + -20.171007xGA + 0.862044xGA^{2} + -0.010213xGA^{3}$
		4186	SD	$8.181534 + -34.670966xGA^{-0.5}$
	**DGMV**	4189	Mean	$2.565062 + -0.905458xGA^{0.5} + 0.000642xGA^{3}$
		4189	SD	$-3.357278 + 0.134731xGA$
	**CBV**	4181	Mean	$20.062157 + -26.210291xLN(GA) + 13.249654xGA^{0.5}$
		4181	SD	$-4.802530 + 0.144062xGA$
	**CPV**	4203	Mean	$-8.747354 + 1.226594xGA + -0.052478xGA^{2} + 0.000745xGA^{3}$
		4203	SD	$-4.017505 + -1353.340000xGA^{-2} + 110.794500xGA^{-1}$
	**LVV**	4203	Mean	$0.199152 + -759.201300xGA^{-3} + 0.000006xGA^{3}$
		4203	SD	$-8.870769 + 1.868799xLN(GA)$
	**FHV**	4201	Mean	$1.957187 + -0.247137xGA + 0.010655xGA^{2} + -0.000135xGA^{3}$
		4201	SD	$-28.094510 + 1841.363000xGA^{-1} + 334027.300000xGA^{-3} + -43795.430000xGA^{-2}$
	**BSV**	4193	Mean	$27.563862 + -187.829409xGA^{-0.5} + 9843.864509xGA^{-2} + -77842.728897xGA^{-3}$
		4193	SD	$41.292163 + -7.207325xLN(GA) + -745.053409xGA^{-1} + 5815.331813xGA^{-2}$

	**THV**	4197	Mean	$-0.006779 + 0.000063xGA^{3}$
		4197	SD	$2.222630 + -21.555120xGA^{-0.5}$
	**CSPV**	4192	Mean	$4.356401 + -0.607443xGA + 0.026988xGA^{2} + -0.000357xGA^{3}$
		4192	SD	$-7.132420 + 0.186246xGA$
**Cortical properties** (mm)	**CoPSA** (cm^2)	4186	Mean	$597.853200 + -84.471030xGA + 3.990118xGA^{2} + -0.053964xGA^{3}$
		4186	SD	$2.844081 + -5768.336000xGA^{-3}$
	**SFD**	4183	Mean	$3.892893 + 0.000398xGA^{3}$
		4183	SD	$-27.308230 + 390.145500xGA^{-0.5} + -1508.030000xGA^{-1} + 6043.224000xGA^{-2}$
	**FLV**	4179	Mean	$-486.864000 + 836.743400xGA^{-0.5} + 100.843200xLN(GA)$
		4179	SD	$11.879910 + -2.004846xGA + 0.099576xGA^{2} + -0.001558xGA^{3}$
	**PLV**	4175	Mean	$72.546200 + -4072.974630xGA^{-1} + 79853.099420xGA^{-2} + -533624.290650xGA^{-3}$
		4175	SD	$19.605890 + -1192.724050xGA^{-1} + 23061.053550xGA^{-2} + -149919.950180xGA^{-3}$
	**OLV**	4179	Mean	$-0.383232 + 0.000181xGA^{3}$
		4179	SD	$37.275040 + -2669.345000xGA^{-1} + 61598.870000xGA^{-2} + -471122.300000xGA^{-3}$
	**TLV**	4182	Mean	$-103.073684 + 438.484975xGA^{-1} + 27.482065xLN(GA)$
		4182	SD	$-0.516115 + -5792.850915xGA^{-3}$
	**ILV**	4183	Mean	$-20.933801 + 5.525688xLN(GA) + 90.762877xGA^{-1}$
		4183	SD	$-0.906794 + -930.595844xGA^{-2}$
	**FLD**	4180	Mean	$-97.005380 + 50259.890000xGA^{-2} + -559464.100000xGA^{-3} + 2.337089xGA$
		4180	SD	$3.114818 + -5482.035000xGA^{-2} + 81529.700000xGA^{-3}$
	**PLD**	4171	Mean	$71.544610 + -3874.344000xGA^{-1} + 79162.820000xGA^{-2} + -561055.100000xGA^{-3}$
		4171	SD	$2.012193 + -0.013391xGA^{2} + 0.000371xGA^{3}$
	**OLD**	4186	Mean	$-104.374200 + 14.495390xGA + -0.638189xGA^{2} + 0.009510xGA^{3}$
		4186	SD	$-13.765670 + 2.312154xGA + -0.121390xGA^{2} + 0.002024xGA^{3}$
	**TLD**	4178	Mean	$3.563134 + 0.000188xGA^{3}$
		4178	SD	$5.248537 + 0.002623xGA^{2} + -2.235585xLN(GA)$
	**ILD**	4185	Mean	$57.005525 + -26.379179xGA^{0.5} + 3.397341xGA$
		4185	SD	$41.294879 + 3.959552xGA + -0.028730xGA^{2} + -24.459888xGA^{0.5}$
	**FLT**	4175	Mean	$568.687760 + -1817.085706xGA^{-0.5} + -91.086921xLN(GA) + 2254.872431xGA^{-1}$
		4175	SD	$306.210455 + -485.211082xLN(GA) + 269.748356xGA^{0.5} + -0.152583xGA^{2}$
	**PLT**	4173	Mean	$17.999250 + -965.055520xGA^{-1} + 19284.531860xGA^{-2} + -126849.193410xGA^{-3}$
		4173	SD	$26.131680 + -1650.634110xGA^{-1} + 30243.942690xGA^{-2} + -171047.217590xGA^{-3}$
	**OLT**	4174	Mean	$3.761465 + -52.382060xGA^{-1} + 7278.347000xGA^{-3}$
		4174	SD	$15.094290 + -1078.198000xGA^{-1} + 20354.610000xGA^{-2} + -109989.600000xGA^{-3}$

	**TLT**	4187	Mean	$2.408148 + -2103.584000xGA^{-3} + 0.000013xGA^{3}$
		4187	SD	$10.535880 + -1.627659xGA + 0.071965xGA^{2} + -0.001101xGA^{3}$
	**ILT**	4179	Mean	$2.157454 + 0.000063xGA^{3} + -3428.151000xGA^{-3}$
		4179	SD	$38.462670 + -2734.983000xGA^{-1} + 62367.260000xGA^{-2} + -475491.300000xGA^{-3}$
	**rPLV**	4196	Mean	$16.108720 + -181.272317xGA^{-0.5} + -16946.099874xGA^{-3} + 536.384239xGA^{-1}$
		4196	SD	$-5.399054 + 30.713968xGA^{-1}$
	**rILV**	4199	Mean	$-0.225281 + 17.421454xGA^{-1} + 2478.884807xGA^{-3} + -372.699098xGA^{-2}$
		4199	SD	$120.122864 + -0.091338xGA^{2} + 11.859717xGA + -72.979961xGA^{0.5}$
	**rILV/rPLV**	4186	Mean	$-15.444110 + 196.854200xGA^{-0.5} + -693.133700xGA^{-1} + 2467.930000xGA^{-2}$
		4186	SD	$-6.391111 + 0.012498xGA^{2} + -0.000333xGA^{3}$

Supplementary Table 9– Cluster table summarising how the magnetic resonance imaging (MRI) cortical parcellation atlas labels were combined for comparison with the ultrasound-derived cortical labels: frontal lobe (FL), temporal lobe (TL), parietal lobe (PL), occipital lobe (OL) and insular lobe (IL). The two MRI atlases, Computational Radiology Lab (CRL) (Gholipour, 2017) and Fetal Brain Atlas (FBA) (Wu, 2021), used the same cortical parcellation map.

	CRL (Gholipour, 2017) and FBA (Wu, 2021)
FL	Precentral_L
	Precentral_R
	Frontal_Sup_L
	Frontal_Sup_R
	Frontal_Sup_Orb_L
	Frontal_Sup_Orb_R
	Frontal_Mid_L
	Frontal_Mid_R
	Frontal_Mid_Orb_L
	Frontal_Mid_Orb_R
	Frontal_Inf_Oper_L
	Frontal_Inf_Oper_R
	Frontal_Inf_Tri_L
	Frontal_Inf_Tri_R
	Frontal_Inf_Orb_L
	Frontal_Inf_Orb_R
	Supp_Motor_Area_L
	Supp_Motor_Area_R
	Frontal_Sup_Medial_L
	Frontal_Sup_Medial_R
	Frontal_Med_Orb_L
	Frontal_Med_Orb_R
	Rectus_L
	Rectus_R
	Cingulum_Ant_L
	Cingulum_Ant_R
	Paracentral_Lobule_L
	Paracentral_Lobule_R
PL	Rolandic_Oper_L
	Rolandic_Oper_R
	Cingulum_Mid_L
	Cingulum_Mid_R
	Cingulum_Post_L
	Cingulum_Post_R
	Postcentral_L
	Postcentral_R

	Parietal_Sup_L<br>Parietal_Sup_R<br>Parietal_Inf_L<br>Parietal_Inf_R<br>SupraMarginal_L<br>SupraMarginal_R<br>Angular_L<br>Angular_R<br>Precuneus_L<br>Precuneus_R
TL	ParaHippocampal_L<br>ParaHippocampal_R<br>Fusiform_L<br>Fusiform_R<br>Heschl_L<br>Heschl_R<br>Temporal_Sup_L<br>Temporal_Sup_R<br>Temporal_Pole_Sup_L<br>Temporal_Pole_Sup_R<br>Temporal_Mid_L<br>Temporal_Mid_R<br>Temporal_Pole_Mid_L<br>Temporal_Pole_Mid_R<br>Temporal_Inf_L<br>Temporal_Inf_R
OL	Calcarine_L<br>Calcarine_R<br>Cuneus_L<br>Cuneus_R<br>Lingual_L<br>Lingual_R<br>Occipital_Sup_L<br>Occipital_Sup_R<br>Occipital_Mid_L<br>Occipital_Mid_R<br>Occipital_Inf_L<br>Occipital_Inf_R
IL	Insula_R<br>Insula_L

Supplementary Table 10- Demographic information about the training and testing dataset .

	**Train**		**Test**	
**Visible Hemisphere**	**Left**	**Right**	**Left**	**Right**
**Newborn Sex**				
Male	70	111	17	22
Female	49	104	11	20
**Study site**				
Beijing, China	19	43	5	8
Muscat, Oman	25	42	5	9
Nagpur, India	18	23	3	3
Nairobi, Kenya	14	31	3	10
Oxford, UK	16	29	4	3
Pelotas, Brazil	8	14	1	3
Seattle, USA	7	11	1	2
Turin, Italy	12	22	6	4
**Gestational week**				
18	25	25	5	6
19	16	17	1	6
20	15	30	3	7
21	14	24	4	4
22	8	15	1	4
23	12	37	4	6
24	10	19	5	1
25	6	20	0	5
26	13	28	5	3

Supplementary Table 11 – Summary of the magnetic resonance imaging (MRI) atlases used for label comparison.

	Gestational age range (weeks)	Regions of interest	Public link
Computational Radiology Lab (CRL) (Gholipour, 2017)	21-38	124	http://crl.med.harvard.edu/research/fetal_brain_atlas/
King's College, London (KCL) (Uus, 2023)	21-36	19	https://gin.gnode.org/kcl_cdb/055t_fetal_mri_atlases
Serag (Serag, 2012)	23-37	4	https://brain-development.org/brain-atlases/fetal-brain-atlases/fetal-brain-atlas-serag/
Chinese (CHN) (Xu, 2022)	23-38	85	https://github.com/Thea-Eddie-Amy/CHN-fetal-brain-atlas/tree/main
Fetal Brain Atlas (FBA) (Wu, 2021)	21-35	124	https://github.com/DeepBMI/FBA-Chinese/tree/main

Supplementary Table 12 – Cluster table summarising how the magnetic resonance imaging atlas (MRI) labels were combined for comparison with the ultrasound atlas labels: total brain volume (TBV), cortical plate volume (CoPV), white matter volume (WMV), deep grey matter volume (DGMV), cerebellum volume (CBV), thalamus volume (ThV), lateral posterior ventricle horns volume (LVV), choroid plexus volume (ChPV), frontal horns volume (FHV), brainstem volume (BSV), cavum septum volume (CSPV). The label names have been kept consistent with each MRI atlas documentation to enable readers to reproduce our results. More information on the datasets is shown in Suppl. Table 8.

	CRL<br>(Gholipour, 2017)	KCL<br>(Uus, 2023)	Serag<br>(Serag, 2012)	CHN<br>(Xu, 2022)	FBA<br>(Wu, 2021)
CoP	Cortical_Plate_L<br>Cortical_Plate_R	Cortical GM Left<br>Cortical GM Right	CoP	Cortical grey matter	Cortical_Plate_L<br>Cortical_Plate_R
CSP		Cavum septum pellucidum			
CB	Cerebellum_L<br>Cerebellum_R	Cerebellum Left<br>Cerebellum Right<br>Cerebellar Vermis		Cerebellum	Cerebellum_L<br>Cerebellum_R
VS	Lateral_Ventricle_R<br>Lateral_Ventricle_L	Lateral Ventricle Left<br>Lateral Ventricle Right	VS	Ventricles	Lateral_Ventricle_R<br>Lateral_Ventricle_L
DGM	Hippocampus_L<br>Hippocampus_R<br>Putamen_L<br>Putamen_R<br>Internal_Capsule_R<br>Internal_Capsule_L<br>Hippocampal_Comm<br>Caudate_L<br>Caudate_R<br>Subthalamic_Nuc_L<br>Subthalamic_Nuc_R<br>Amygdala_L<br>Amygdala_R	Basal Ganglia Left<br>Basal Ganglia Right<br>Third Ventricle<br>Fourth Ventricle		Deep Grey Matter<br>Hippocampi and Amygdala	Hippocampus_L<br>Hippocampus_R<br>Putamen_L<br>Putamen_R<br>Internal_Capsule_R<br>Internal_Capsule_L<br>Hippocampal_Comm<br>Caudate_L<br>Caudate_R<br>Subthalamic_Nuc_L<br>Subthalamic_Nuc_R<br>Amygdala_L<br>Amygdala_R
Th	Thalamus_L<br>Thalamus_R	Thalamus Left<br>Thalamus Right			Thalamus_L<br>Thalamus_R
BS	Midbrain_L	Brainstem		Brainstem	Midbrain_L
WM	Subplate_L<br>Inter_Zone_L<br>Vent_Zone_L<br>Subplate_R<br>Inter_Zone_R<br>Vent_Zone_R	Fetal WM Left<br>Fetal WM Right		White matter	Subplate_L<br>Inter_Zone_L<br>Vent_Zone_L<br>Subplate_R<br>Inter_Zone_R<br>Vent_Zone_R
