# Supplementary material for: Normative growth trajectories of fetal brain regions validated by satisfactory maturation of neurodevelopmental domains at 2 years of age
Source: Nat Commun. 2026 Feb 23;17:3073. doi: 10.1038/s41467-026-69657-w (PMC13040065; doi:10.1038/s41467-026-69657-w)
Supplement: Supplementary file 2 — Reporting Summary [file 41467_2026_69657_MOESM2_ESM.pdf]

nature portfolio

Corresponding author(s): Madeleine Wyburd

Last updated by author(s): Jul 21, 2025

Reporting Summary

Nature Portfolio wishes to improve the reproducibility of the work that we publish. This form provides structure for consistency and transparency in reporting. For further information on Nature Portfolio policies, see our Editorial Policies and the Editorial Policy Checklist.

Statistics

For all statistical analyses, confirm that the following items are present in the figure legend, table legend, main text, or Methods section.

n/a	Confirmed	
☐	☒	The exact sample size (*n*) for each experimental group/condition, given as a discrete number and unit of measurement
☐	☒	A statement on whether measurements were taken from distinct samples or whether the same sample was measured repeatedly
☒	☐	The statistical test(s) used AND whether they are one- or two-sided<br>*Only common tests should be described solely by name; describe more complex techniques in the Methods section.*
☐	☒	A description of all covariates tested
☐	☒	A description of any assumptions or corrections, such as tests of normality and adjustment for multiple comparisons
☐	☒	A full description of the statistical parameters including central tendency (e.g. means) or other basic estimates (e.g. regression coefficient) AND variation (e.g. standard deviation) or associated estimates of uncertainty (e.g. confidence intervals)
☒	☐	For null hypothesis testing, the test statistic (e.g. *F*, *t*, *r*) with confidence intervals, effect sizes, degrees of freedom and *P* value noted<br>*Give P values as exact values whenever suitable.*
☒	☐	For Bayesian analysis, information on the choice of priors and Markov chain Monte Carlo settings
☒	☐	For hierarchical and complex designs, identification of the appropriate level for tests and full reporting of outcomes
☒	☐	Estimates of effect sizes (e.g. Cohen's *d*, Pearson's *r*), indicating how they were calculated

Our web collection on statistics for biologists contains articles on many of the points above.

Software and code

Policy information about availability of computer code

Data collection	No specialised software was used for data collection
Data analysis	The complete data analysis pipeline is available here: https://oxford-omni-lab-org.github.io/OMNI_ultrasound. All ultrasound processing was performed on Python 3.11, with the deep-learning implementation using Pytorch 2.1.0. Statistical analysis was performed in R-Studio (version 2024.04.2+764) using GAMLSS (version 5.4-22).

For manuscripts utilizing custom algorithms or software that are central to the research but not yet described in published literature, software must be made available to editors and reviewers. We strongly encourage code deposition in a community repository (e.g. GitHub). See the Nature Portfolio guidelines for submitting code & software for further information.

Data

Policy information about availability of data

All manuscripts must include a data availability statement. This statement should provide the following information, where applicable:

- Accession codes, unique identifiers, or web links for publicly available datasets
- A description of any restrictions on data availability
- For clinical datasets or third party data, please ensure that the statement adheres to our policy

The labeled ultrasound atlases will be made publicly available on https://intergrowth21.com/research/brain-atlas-project. The derived growth curves are available in the Supplementary materials. Owing to the data still being under analysis for the principal and secondary objectives of the study protocol, anonymized image data will be made available on reasonable request for academic use only and within the limitations of the informed consent. Requests must be made to the

corresponding author or to the INTERGROWTH-21st Consortium at intergrowth@wrh.ox.ac.uk. Full conditions of access are available in the INTERGROWTH-21st study protocol at https://intergrowth21.com/research/brain-atlas-project. Every request will be reviewed by the INTERGROWTH-21st Consortium Executive Committee with due promptness. After approval, the researcher will need to sign a data access agreement with the INTERGROWTH-21st Consortium.

Research involving human participants, their data, or biological material

Policy information about studies with human participants or human data. See also policy information about sex, gender (identity/presentation), and sexual orientation and race, ethnicity and racism.

Reporting on sex and gender	The participating mothers are all female, and their newborn babies are described as male or female.
Reporting on race, ethnicity, or other socially relevant groupings	The study did not collect any information about ethnicity or ancestral background
Population characteristics	The study was population-based and conducted in eight delimited urban areas: Turin (Italy), Oxford (UK), Pelotas (Brazil), Nairobi (Kenya), Muscat (Oman), Beijing (China) and Nagpur (India).
Recruitment	Participating women, who initiated antenatal care before 14 weeks' gestation, were selected based on WHO criteria for optimal health, nutrition, education and socioeconomic status needed to construct internal growth standards.
Ethics oversight	The INTERGROWTH-21st Project and its ancillary studies were approved by the Oxfordshire Research Ethics Committee "C" (reference: 08/H0606/139), the research ethics committees of the individual participating institutions, as well as the corresponding regional health authorities where the project was implemented.

Note that full information on the approval of the study protocol must also be provided in the manuscript.

Field-specific reporting

Please select the one below that is the best fit for your research. If you are not sure, read the appropriate sections before making your selection.

☒ Life sciences ☐ Behavioural & social sciences ☐ Ecological, evolutionary & environmental sciences

For a reference copy of the document with all sections, see nature.com/documents/nr-reporting-summary-flat.pdf

Life sciences study design

All studies must disclose on these points even when the disclosure is negative.

Sample size	The sample size of the original fetal growth study (Lancet 2014) was based on pragmatic and statistical considerations; the latter focused on the precision and accuracy of a single extreme centile, i/e the 3rd or 97th centile, and regression based reference limits. The availability of fetal brain volumes from the original study determined the sample to be analysed here.
Data exclusions	We excluded 765 subjects who were missing follow-up at 1 or 2 years of age, or with severe morbidity detected at the same age intervals. We further excluded 101 subjects who scored in the bottom 3% of any INTER-NDA domain at age 2. This exclusion is shown in Fig1a
Replication	To verify our structure labeling on the ultrasound atlas, we compared the volume measures of 14 structures to previous MRI atlases and found a strong overlap (Fig.4) . Additionally, we compared the outputs of the segmentation models to a popular MRI based model and found highly correlated and overlapping volume measures (Wyburd et al. 2025). We performed leave-one-out-experiments on each site and found the derived curves remained the same when excluded each site (Fig 5a). Finally, the segmentation code is publicly available to enable other researches to replicate our findings.
Randomization	N/A
Blinding	The team analysing the fetal brain volumes were blinded to the infant neurodevelopmental outcomes.

Reporting for specific materials, systems and methods

We require information from authors about some types of materials, experimental systems and methods used in many studies. Here, indicate whether each material, system or method listed is relevant to your study. If you are not sure if a list item applies to your research, read the appropriate section before selecting a response.

Materials & experimental systems

n/a	Involved in the study
☒	☐ Antibodies
☒	☐ Eukaryotic cell lines
☒	☐ Palaeontology and archaeology
☒	☐ Animals and other organisms
☒	☐ Clinical data
☒	☐ Dual use research of concern
☒	☐ Plants

Methods

n/a	Involved in the study
☒	☐ ChIP-seq
☒	☐ Flow cytometry
☒	☐ MRI-based neuroimaging

Plants

Seed stocks

Report on the source of all seed stocks or other plant material used. If applicable, state the seed stock centre and catalogue number. If plant specimens were collected from the field, describe the collection location, date and sampling procedures.

Novel plant genotypes

Describe the methods by which all novel plant genotypes were produced. This includes those generated by transgenic approaches, gene editing, chemical/radiation-based mutagenesis and hybridization. For transgenic lines, describe the transformation method, the number of independent lines analyzed and the generation upon which experiments were performed. For gene-edited lines, describe the editor used, the endogenous sequence targeted for editing, the targeting guide RNA sequence (if applicable) and how the editor was applied.

Authentication

Describe any authentication procedures for each seed stock used or novel genotype generated. Describe any experiments used to assess the effect of a mutation and, where applicable, how potential secondary effects (e.g. second site T-DNA insertions, mosiacism, off-target gene editing) were examined.
